# Watershed Urbanization Linked to Differences in Stream Bacterial Community Composition

**DOI:** 10.3389/fmicb.2017.01452

**Published:** 2017-08-02

**Authors:** Jacob D. Hosen, Catherine M. Febria, Byron C. Crump, Margaret A. Palmer

**Affiliations:** ^1^Chesapeake Biological Laboratory Solomons, MD, United States; ^2^Department of Entomology, University of Maryland College Park, MD, United States; ^3^College of Earth, Ocean, and Atmospheric Sciences, Oregon State University Corvallis, OR, United States; ^4^School of Biological Sciences, University of Canterbury Christchurch, New Zealand; ^5^School of Forestry and Environmental Studies, Yale University New Haven, CT, United States; ^6^National Socio-Environmental Synthesis Center Annapolis, MD, United States

**Keywords:** 16S rRNA gene, bacteria, community ecology, streams, urbanization, watersheds

## Abstract

Urbanization strongly influences headwater stream chemistry and hydrology, but little is known about how these conditions impact bacterial community composition. We predicted that urbanization would impact bacterial community composition, but that stream water column bacterial communities would be most strongly linked to urbanization at a watershed-scale, as measured by impervious cover, while sediment bacterial communities would correlate with environmental conditions at the scale of stream reaches. To test this hypothesis, we determined bacterial community composition in the water column and sediment of headwater streams located across a gradient of watershed impervious cover using high-throughput 16S rRNA gene amplicon sequencing. Alpha diversity metrics did not show a strong response to catchment urbanization, but beta diversity was significantly related to watershed impervious cover with significant differences also found between water column and sediment samples. Samples grouped primarily according to habitat—water column vs. sediment—with a significant response to watershed impervious cover nested within each habitat type. Compositional shifts for communities in urbanized streams indicated an increase in taxa associated with human activity including bacteria from the genus *Polynucleobacter*, which is widespread, but has been associated with eutrophic conditions in larger water bodies. Another indicator of communities in urbanized streams was an OTU from the genus *Gallionella*, which is linked to corrosion of water distribution systems. To identify changes in bacterial community interactions, bacterial co-occurrence networks were generated from urban and forested samples. The urbanized co-occurrence network was much smaller and had fewer co-occurrence events per taxon than forested equivalents, indicating a loss of keystone taxa with urbanization. Our results suggest that urbanization has significant impacts on the community composition of headwater streams, and suggest that processes driving these changes in urbanized water column vs. sediment environments are distinct.

## Introduction

Understanding the patterns and drivers of biodiversity is central to predicting ecosystem responses to environmental change. This is particularly true for microbes because of the key roles they play in global biogeochemical cycles. Despite recent advances in sequencing technologies, identifying the mechanisms that underlie microbial diversity remains a major challenge. This challenge is particularly significant for highly dynamic ecosystems such as flowing-waters where temporal and spatial variability in flows are often dramatic (Poff et al., 2006). These environments host mosaics of habitat patches including surface and subsurface water, sediment, and epilithic biofilms that differ in their environmental conditions (Winemiller et al., 2010) and set the stage for local adaptation and patch scale species sorting by microbes (Adams et al., 2014). However, the pool of dispersing microbes available to colonize these patches is highly dynamic (Zeglin, 2015)—for example, bacteria can be suspended into stream water following streambed disturbances and many microbes appear to enter stream water from watershed sources (Crump et al., 2003, 2007, 2012).

The combination of high habitat heterogeneity and a large dispersal potential in running-water systems has led researchers to suggest that frameworks from landscape ecology and metacommunity theory may be useful in studies of the diversity and composition of stream microbial communities (Battin et al., 2007). Specifically, water column bacteria represent a pool of microbes available to colonize benthic habitats after which local adaptation and patch-scale species sorting can occur; both dispersal and local environmental conditions may influence microbial diversity, and composition albeit at different proportions in different habitat types (Crump et al., 2007, 2012; Besemer et al., 2013). For microbes in environments with longer residence times and decreased colonization rates, such as stream bed environments, environmental sorting has a stronger influence on microbial composition (Or et al., 2012; Adams et al., 2014; Handley et al., 2014) except in cases where mass effects are strong and continual (Souffreau et al., 2014).

Watershed land use, including urbanization, influences microbial diversity, and composition (Belt et al., 2007; Wang et al., 2011) through both dispersal and by changing the environment at the patch scale. Urbanized landscapes are likely the sources of novel microbial taxa not found in undisturbed stream ecosystems, including taxa from sewage and septic systems, water distribution systems, and stormwater management ponds. Urbanization also changes the local physical and chemical milieu of stream habitat patches by, for example, subjecting stream reaches to sediment erosion or deposition, elevated conductivity, temperature, nutrients, altered organic matter quality, or other stressors (Walsh et al., 2005; Hosen et al., 2014). Flow extremes exacerbated by urban development (Paul and Meyer, 2001) may directly alter the composition of bacterial communities via scouring and dispersal, and indirectly change communities by altering sediment size—as has been shown for denitrifying taxa (Perryman et al., 2011b). These flow-related effects on biological diversity are well-known for larger stream organisms such as macroinvertebrates (Moore and Palmer, 2005; Wenger et al., 2009), but the impact of urbanization on bacterial community composition is less clear.

Most urbanization studies on microbial communities have focused on pathogenic taxa such as fecal coliform bacteria (Nagy et al., 2012; Daly et al., 2013; Kapoor et al., 2014), denitrifying bacteria (Hale and Groffman, 2006; Knapp et al., 2009; Perryman et al., 2011b; Harrison et al., 2012), or unicellular algae and diatoms (Hill et al., 2000; Elsdon and Limburg, 2008). The handful of studies that were not limited to coliforms, denitrifiers, or algae/diatoms have suggested a large difference between bacterial communities in biofilms (Lear and Lewis, 2009; Lear et al., 2011), streambed sediments (Jackson and Weeks, 2008; Perryman et al., 2011a; Wang et al., 2011), and the water column (Belt et al., 2007; Or et al., 2013). This body of work provides important insights, but these studies used relatively coarse measurements of microbial diversity such as denaturing gel gradient electrophoresis (DGGE), automated ribosomal intergenic spacer analysis (ARISA), and terminal restriction fragment length polymorphism (T-RFLP) analysis. The coarse unit of analysis for these fingerprinting techniques is inadequate for testing of alpha diversity (Dunbar et al., 2000), community interactions, and potential functions (Lozupone and Knight, 2007; Hamady and Knight, 2009). Our goal was to determine how urbanization influences stream microbial diversity in both the water column and sediments using a high-throughput sequencing approach that provides higher taxonomic resolution and allows for direct cross-study comparisons.

We used high throughput sequencing of 16S rRNA genes to quantify bacterial community composition in 11 streams (hereafter, “sites”) in watersheds in which the dominant land cover was forest with varying levels of urbanization—as measured by percent watershed impervious cover. To explore potential links between composition and environmental factors we also measured physicochemical parameters known to influence aquatic diversity. Our specific objectives were to: (1) quantify alpha diversity in different habitat types (water column vs. sediment) and landscape urbanization—as determined by percent watershed impervious cover; (2) quantify beta diversity and taxa co-occurrence patterns across habitats and across a gradient of urbanization; and, (3) identify the environmental factors that explain variation in community composition.

Communities of larger stream organisms—including macroinvertebrates and fish—show compositional differences among stream habitat types and across watershed land uses (Morgan et al., 2007; Campbell and McIntosh, 2013). We anticipated similar patterns among microbial communities including large differences between habitat types, and correlations between microbial alpha- and beta-diversity and the magnitude of landscape urbanization. We hypothesized that water column communities are more strongly related to watershed land use than sediment communities because delivery of microbes from the landscape is a major driver of community composition for microbes suspended in stream water columns (Crump et al., 2012). By contrast, we hypothesized that sediment communities are more strongly linked to environmental conditions within stream reaches because these habitats are more stable and bacterial residence time is longer. Thus, we expected to find evidence that mass effects strongly influence community composition in the water column, but that species sorting would mute the impact of mass effects in sediment environments.

## Materials and methods

### Study sites

**Figure 1:**
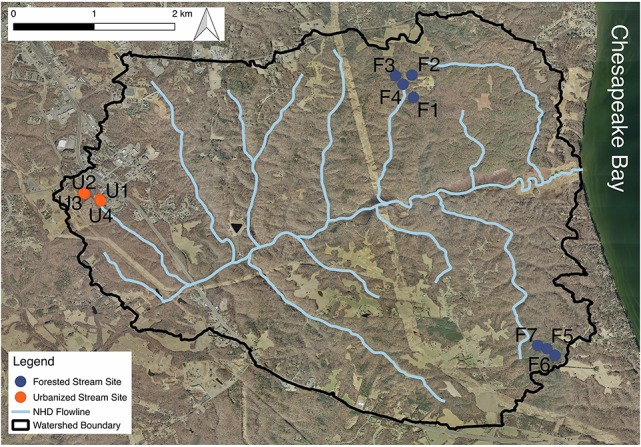
A map of the Parkers Creek watershed with the study sites indicated. Some stream sampling sites are located in channels that are not included in NHD stream maps used. Aerial orthophotography was provided by Calvert County, Maryland government.

The study was conducted in the Parkers Creek watershed, located on the Western Shore of the Chesapeake Bay in the Coastal Plain of Maryland, USA. Study streams were all first and second order. Many of the study streams are unmapped; therefore, stream order was determined using field surveys and watershed orthophotography for the 11 headwater streams reported on in this study (Figure 1). Sites spanned a gradient of land cover urbanization with seven forested (F) headwater stream sites (64.4–100% forested; 0–4.9% impervious), and four urbanized (U) headwater sites (2.5–33.5% forested; 10–44% impervious cover) (Table 1). For most analyses, watershed impervious cover was used as a measure of level of urbanization. For network analysis and analysis of similarity (ANOSIM), we divided sites into two groups—forested and urbanized—based on the substantial difference in impervious cover and forest cover between the two groups. A digital elevation model (DEM) of the Parkers Creek watershed was generated from Light Detection and Ranging (LiDAR) data collected in March 2011 and provided by Calvert County, MD. Field site watersheds were extracted from the DEM using ArcGIS 10.1 (ESRI, Redlands, CA, USA). Impervious and forested land cover were manually delineated with ArcGIS using orthophotography also supplied by Calvert County, MD government.

**Table 1:** Summary of site identities, watershed area, and location.

**Site**	**Primary land cover**	**Stream reach order**	**Watershed area (Hectares)**	**Latitude**	**Longitude**	**% Forest cover**	**% Impervious cover**
**F1**	Forested	First	11.70	38°32′51.31″N	76°32′28.97″W	90.6	0.4
**F2**	Forested	First	4.413	38°33′01.94″N	76°32′30.23″W	64.4	4.9
**F3**	Forested	First	6.970	38°33′01.62″N	76°32′39.14″W	94.7	2.1
**F4**	Forested	First	2.855	38°30′41.87″N	76°31′21.21″W	100	0.0
**F5**	Forested	First	2.778	38°30′38.71″N	76°31′16.67″W	90.8	2.0
**U1**	Urbanized	First	1.530	38°31′58.57″N	76°35′09.51″W	33.5	44.0
**U2**	Urbanized	First	7.708	38°32′01.84″N	76°35′18.19″W	27.7	24.3
**U3**	Urbanized	First	4.693	38°32′00.43″N	76°35′17.90″W	2.5	10.0
**F7**	Forested	Second	16.51	38°30′44.08″N	76°31′25.32″W	93.0	2.5
**F6**	Forested	Second	21.38	38°32′57.27″N	76°32′35.09″W	86.5	1.7
**U4**	Urbanized	Second	27.51	38°31′57.68″N	76°35′09.02″W	24.2	24.1

### Sample collection

Following methods in Crump et al. (2003), water column samples were collected in February, April, August, and November 2012 and February 2013 from all sites with surface water present. Sediment samples were collected in August and November 2012 and in February 2013. In the field, 500 mL of stream water were filtered (Millipore Sterivex-GP 0.22 μm), residual water expelled and ~2 mL of DNA extraction buffer added, after which the filter ports were sealed. Twenty sediment cores were collected randomly from the streambed to a depth of 3 cm (2.67 cm diameter sterile plastic corer) along a 20-m reach at each site on each sampling date; cores at a site were combined in a single sterile Nasco Whirl-Pak bag.

### Environmental parameters

Water temperature, specific conductivity, pH, and dissolved oxygen (DO) were measured with a YSI, Inc. Professional Plus multiparameter meter (Yellow Springs, OH). Samples for dissolved organic carbon (DOC) concentration and DOC quality measurements were filtered in the field with 0.7 μm GF/F filters (Whatman Inc., Maidenstone, UK) and stored in borosilicate amber glass bottles with Teflon-coated lids. Water for other analyses was filtered in the field into amber HDPE plastic bottles. All sample bottles were acid washed in 10% HCl for at least 24 h and both glass bottles and GF/F filters were combusted for 4 h at 450°C. All samples were returned to the laboratory on ice. Sediment subsamples were taken for particulate elemental analysis and grain size analysis. Organic carbon samples were kept at 4°C and were analyzed within 72 h of collection. Samples for genetic analysis were stored at −80°C prior to processing. Other samples were frozen until processing at a later date.

Total DOC, as non-purgeable organic carbon, and total dissolved nitrogen (TDN) were determined by analysis on a Shimadzu TOC-vCPH with attached TNM-1 unit (Shimadzu Corporation; Kyoto, Japan). Carbon quality was determined using the fluorescence index (FI), which indicates if dissolved organic matter (DOM) is primarily allochthonous or autochthonous (McKnight et al., 2001), and the humification index (HIX), which measures the amount of humic DOM (Zsolnay et al., 1999). Fluorescence data were collected on a Horiba Scientific Fluoromax-4 as described previously (Hosen et al., 2014). The fluorescence index was determined as the ratio of fluorescence emission intensities at 450 and 500 nm when a water sample was excited at 370 nm (McKnight et al., 2001). Humification index (HIX) values were determined as the ratio of the area of the emission spectrum at 435–480 nm to the emission area from 300 to 445 nm at an excitation wavelength of 255 nm, (Zsolnay et al., 1999; Plaza et al., 2009; Williams et al., 2010). Dissolved organic nitrogen (DON) was calculated by subtracting dissolved inorganic nitrogen (DIN), ammonium, and nitrate from TDN concentrations. DIN was defined as the sum of dissolved ammonium and nitrate. A Lachat QuikChem 8500 Series 2 flow injection analyzer was used to obtain dissolved nitrate, ammonium, and orthophosphate concentrations. Sediment particulate carbon and nitrogen content were determined using a Costech ECS-4010 elemental analyzer (Costech Analytical Technologies, Valencia, CA).

Sediment samples were dried until weight was constant for 24 h, and then were passed through 2, 1 mm, 500, and 250 μm sieves. The mass of each sediment size fraction was used to determine D90—the 90th percentile sediment particle size, measured in mm—using the R package G2Sd (Folk and Ward, 1957; Fournier et al., 2014).

### Genetic sampling and processing

Water column microbial DNA was extracted from Sterivex-GP filters using phenol-chloroform based on established protocols (Crump et al., 2003). Filters with DNA extraction buffer were defrosted, removed from capsules, and 20 μL of 1% proteinase-K and 20 μL of 10% lysozyme was added to each filter. Samples were frozen at −80°C for 15 min and then thawed at 37°C for 5 min three times. Samples were then incubated in a water bath for 37°C for 30 min. Fifty microliters of 20% filter-sterilized sodium dodecyl sulfate were added to each sample before a 2-h incubation in a 65°C water bath. Samples were washed twice with buffered phenol-chloroform-isoamyl alcohol and then precipitated at room temperature overnight by adding isopropyl alcohol at 60% of sample volume. Sediment DNA was extracted using PowerSoil DNA Isolation Kits (Mo Bio Laboratories, Inc., Carlsbad, CA) and 0.5 g of each sediment sample. PCR amplicons were produced using standard methods for high-throughput sequencing (Caporaso et al., 2012). Amplification of 16S rRNA genes was conducted using forward primer 515f and barcoded reverse primer 806r (Caporaso et al., 2011). For each sample 12 μL of UV-sterilized PCR-grade water, 10 μL 5-prime HotMasterMix, 1 μL 5 mM forward primer, 1 μL of 5 mM reverse primer, and 1 μL of template DNA were combined in a 96-well PCR plate. Conditions for PCR were as follows: initial denaturation for 3 min at 94°C followed by 30 cycles first at 94°C for 0.75 min, 50°C for 1 min, and 72°C for 1.5 min. At the conclusion of each PCR run, temperature was held at 72°C for 10 min before temperature was reduced to 10°C. Amplicons were quantified with Pico-Green dsDNA quantification kit (Life Technologies; Carlsbad, CA), combined in equimolar quantities, and cleaned using an UltraClean PCR Clean-Up kit (MO BIO Laboratories, Inc; Carlsbad, CA). Illumina MiSeq 2 × 150 bp sequencing was conducted at Argonne National Laboratory (Lemont, IL).

Genetic data were analyzed using the software Quantitative Insights into Microbial Ecology (QIIME). Paired end reads were matched using FLASh (Magoc and Salzberg, 2011). USEARCH 6.1 (Edgar, 2010) was used to identify OTUs at 97% similarity from the Silva 111 database and to identify chimeric sequences. Taxonomy was assigned using the RDP Classifier (Wang et al., 2007) at a threshold of 80%. Sequences were subsequently aligned using PyNAST (Caporaso et al., 2010). Sequences identified as belonging to chloroplasts, mitochondria, and the order Thermales were removed from the dataset. Thermales were removed because the taxa from this extremophilic group were considered to be most likely misidentified or inactive taxa (Ho et al., 2016). Less than 0.001% of all sequences were removed by this filtering step. Each sample was then rarified to 25,000 sequences. The complete rarified OTU table used for analysis is included in Supplementary Datasheet 1. Sequences are available from NCBI under accession number SRP110593.

### Analyses to test hypotheses

Watershed percent impervious cover was used to represent degree of landcover urbanization. To preserve normality for statistical analysis, percent impervious cover was transformed by adding one and taking the base-10 logarithm of the resulting number. All environmental data used in the analyses presented here are available in Supplementary Datasheet 2. Metadata associated with Supplementary Datasheet 2 is provided in Supplementary Table 2.

#### Objective 1. microbial diversity

Bacterial alpha-diversity was assessed with richness, Faith's Phylogenetic Diversity, and Shannon Diversity. Bacterial species richness was estimated using CatchAll (Bunge et al., 2012) on rarified OTU tables. Other diversity metrics were assessed using the R package *vegan* 2.3-4 (Oksanen et al., 2016). The response of bacterial diversity to urbanization was tested by comparing each diversity metric to log-transformed watershed percent impervious cover with habitat type (water column vs. sediment) included as a categorical covariate using analysis of covariance (ANCOVA) carried out using the package *nlme* (Pinheiro et al., 2016) in R 3.3.1 (R Core Team, 2016).

#### Objective 2. community composition and beta diversity

Beta-diversity was assessed using principal coordinate analysis (PCoA) of community dissimilarity matrices calculated as Bray-Curtis distances using the R package *vegan* 2.3-4 (Oksanen et al., 2016) and weighted and unweighted Unifrac distances computed in QIIME (Lozupone et al., 2011). To determine dissimilarity across a gradient of watershed impervious land cover and between bacterial communities in sediment and water column habitats, *adonis*—a permutational MANOVA test calculated with the *vegan* package in R (Oksanen et al., 2016)—was conducted with habitat type as a categorical variable and log-transformed watershed impervious cover as a continuous variable. For ANOSIM, community similarity between four sample groups (water column/urbanized, water column/forested, sediment/urbanized, and sediment/forested) was tested using the R package *vegan*. To avoid inflation of type I error, permutations for both *adonis* and ANOSIM were restricted to reflect the repeated measurements of sites and the lack of independence between sediment and water column samples (Edgington and Onghena, 2007). Specifically, permutations were blocked by site, meaning that samples from an individual site were always shuffled together. Further, shuffling between water column and sediment samples was also restricted because of the paired nature of these two sample types. For each permutation test, 1,000 permutations were applied.

To identify the taxa driving differences in community composition between the four categories of sample type, indicator species analysis was applied. Indicator species analysis identifies taxa that are representative of samples coming from distinct habitat groups (Fortunato et al., 2013). If an indicator value for a particular group is greater than 0.3 and that taxon has a significant *p*-value (α = 0.05), then that taxon is considered an indicator. Indicator species analysis was conducted using the *indicspecies* package (Caceres and Legendre, 2009) in R 3.3.1.

Microbial co-occurrence networks allow researchers to understand how and to what extent taxa co-occur within individual communities as well as to identify likely keystone taxa in ecosystems (Barberán et al., 2011; Lupatini et al., 2014; Widder et al., 2014; Williams et al., 2014) such as those in our forested and urban stream sites. We executed our network analysis using the CoNet 1.1.1 plugin for Cytoscape 3.4.0 following established methods (Faust et al., 2012, 2015): Network relationships were calculated from four measures—Bray-Curtis similarity, Kullback-Leibler divergence, and Pearson and Spearman Correlation. OTUs with fewer than 20 sequences within a dataset were excluded from analysis. Null distributions of all pair-wise scores were generated from 1,000 iterations of each dataset. Significance thresholds for each of the four measures of correlation or similarity were set to include the top 5% of pair-wise scores. Brown's method (Brown, 1975) was applied to merge *p*-values from each of the four measures and corrections for multiple tests were applied following Benjamini and Hochberg (1995) with a threshold *p*-value of 0.05. To confirm that the network generated was not the product of random correlations, a comparison was made with randomly generated networks following (Lupatini et al., 2014).

Network statistics are sensitive to the number of samples used for network construction (Faust et al., 2015). There were almost twice as many samples collected from forested sites as there were from urbanized sites. To ensure comparable networks, forested, and urbanized networks were constructed using 25 samples each. To generate the forested network, 100 bootstrapped networks were generated from 25 samples each. The 100 subsampled datasets were generated by randomly selecting 15 forested water column samples and 10 forested sediment samples in order to match the ratio of sediment to water column samples found in the urbanized network. These networks were then merged by using edges that were found in greater than 50%—in this case at least 51—of the bootstrapped networks.

For each node, network centrality metrics including degree, closeness centrality, and betweenness centrality were calculated. These metrics have the potential to identify keystone species within community networks (Williams et al., 2014) with evidence that both node degree and closeness centrality are positively linked to keystone taxa (Berry and Widder, 2014). Differences among network co-occurrence relationships across the two types of networks generated (sediment, water column) were tested using a permutation test described by Williams et al. (2014). Differences in network structures were described by modularity, transitivity, average path length, and average node degree (Newman, 2003, 2006; Barberán et al., 2011). Network statistics were generated using R 3.3.1 with the *igraph* (Csardi and Nepusz, 2006) package and were visualized using the software package Gephi 0.8.2 (Bastian et al., 2009). The forested and urbanized co-occurrence networks produced for this study are available as GraphML files in Supplementary Datasheet 3.

#### Objective 3. environmental factors and community composition

An analysis of covariance (ANCOVA) was applied to assess the relative strength of the link between bacterial community composition and watershed impervious cover in sediment vs. water column samples using principal coordinate axis 1 to represent community composition. Compound symmetry covariance structures were assumed for this analysis. Analysis was conducted using the package *nlme* in R 3.3.1.

The relationship between environmental variables and microbial community structure was assessed using canonical correspondence analysis (CCA) in the *vegan* package. Datasets were divided by habitat (sediment and water column), resulting in two separate models. Based on evaluations of the normality the following environmental variables were log-transformed: discharge, DOC concentration, TDN concentration, FI, sediment C:N, and D90 for this analysis.

## Results

### Microbial diversity

**Figure 2:**
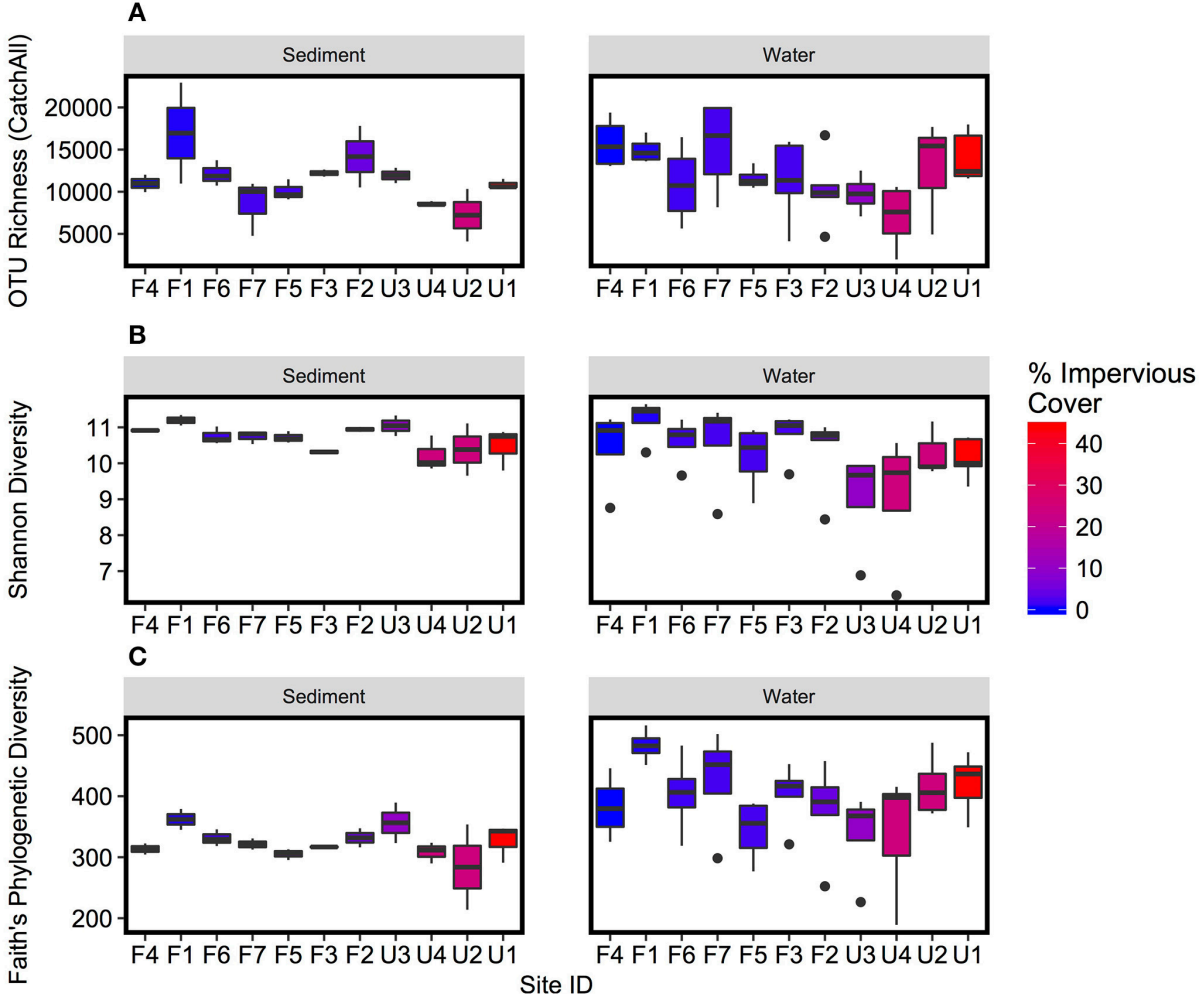
Boxplots of **(A)** OTU richness, as measured by CatchAll, **(B)** Shannon Diversity, and **(C)** Faith's Phylogenetic Diversity of microbial OTUs. Boxplots represent variation over repeated collections of sediment and water samples respectively. Sites are plotted in order of increasing watershed impervious cover. Plots comparing diversity to watershed percent impervious cover are presented in Supplementary Figure 1.

Few significant differences in alpha diversity were detected between sample types (Figure 2), and there were no significant differences for OTU richness measured with CatchAll. Shannon Diversity was negatively correlated with percent watershed impervious cover for both water column and sediment samples (*p* < 0.01; Figure 2B, Supplementary Figure 1B), indicating decreased diversity with increased urbanization. Faith's Phylogenetic Diversity was higher for water column than sediment samples, but there was no significant relationship with watershed impervious cover.

### Community composition and beta diversity

**Figure 3:**
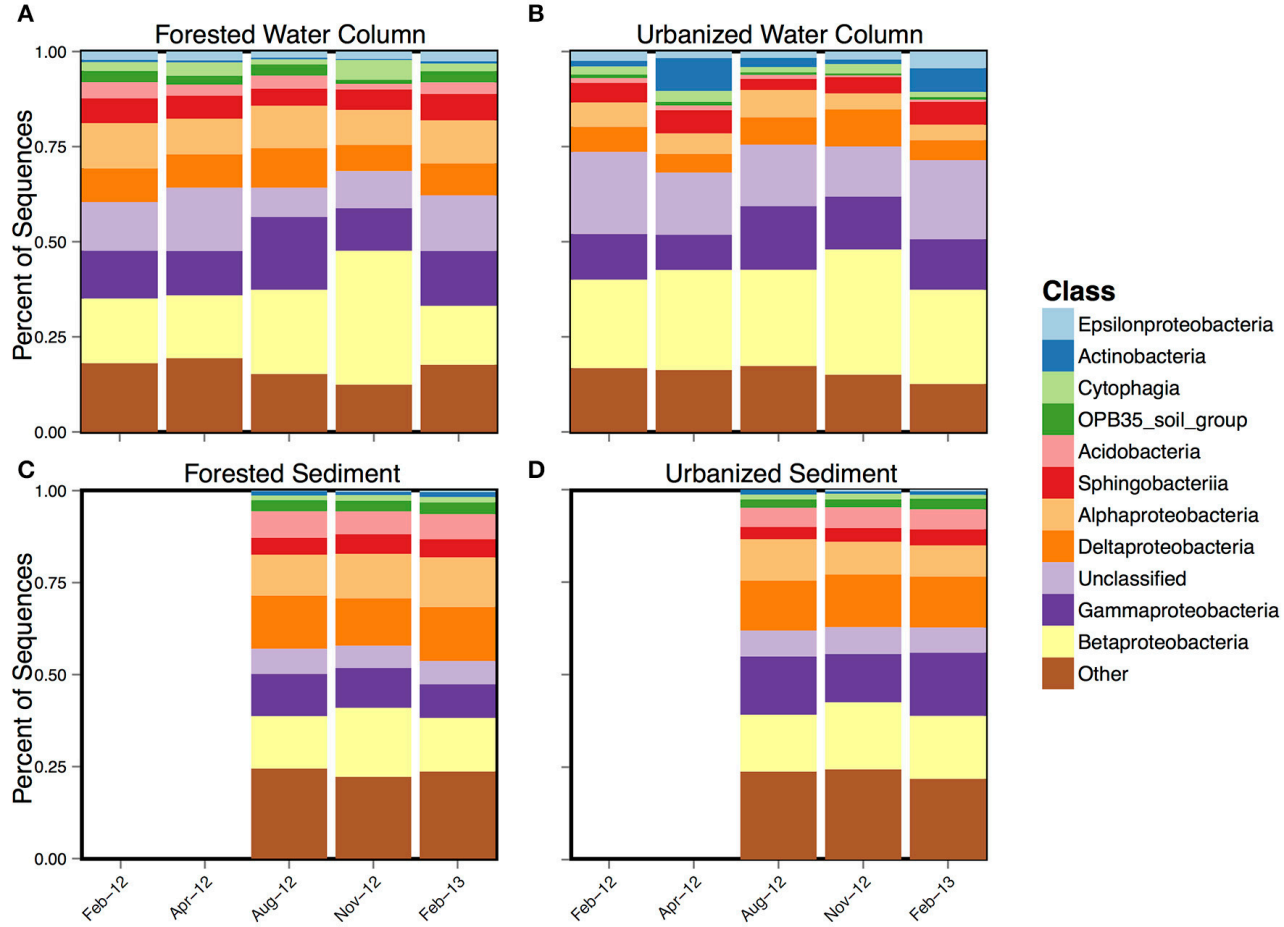
Mean class-level composition over time for **(A)** forested water column, **(B)** forested sediment, **(C)** urbanized water column, and **(D)** urbanized sediment samples.

Taxa from the phylum Proteobacteria dominated all sample types (Figure 3) with either Alphaproteobacteria or Gammaproteobacteria being the most abundant in almost all samples. Urbanized water column samples generally had higher levels of Actinobacteria, particularly in April 2012 and February 2013, and lower levels of Betaproteobacteria and Acidobacteria compared to forested water column samples.

**Figure 4:**
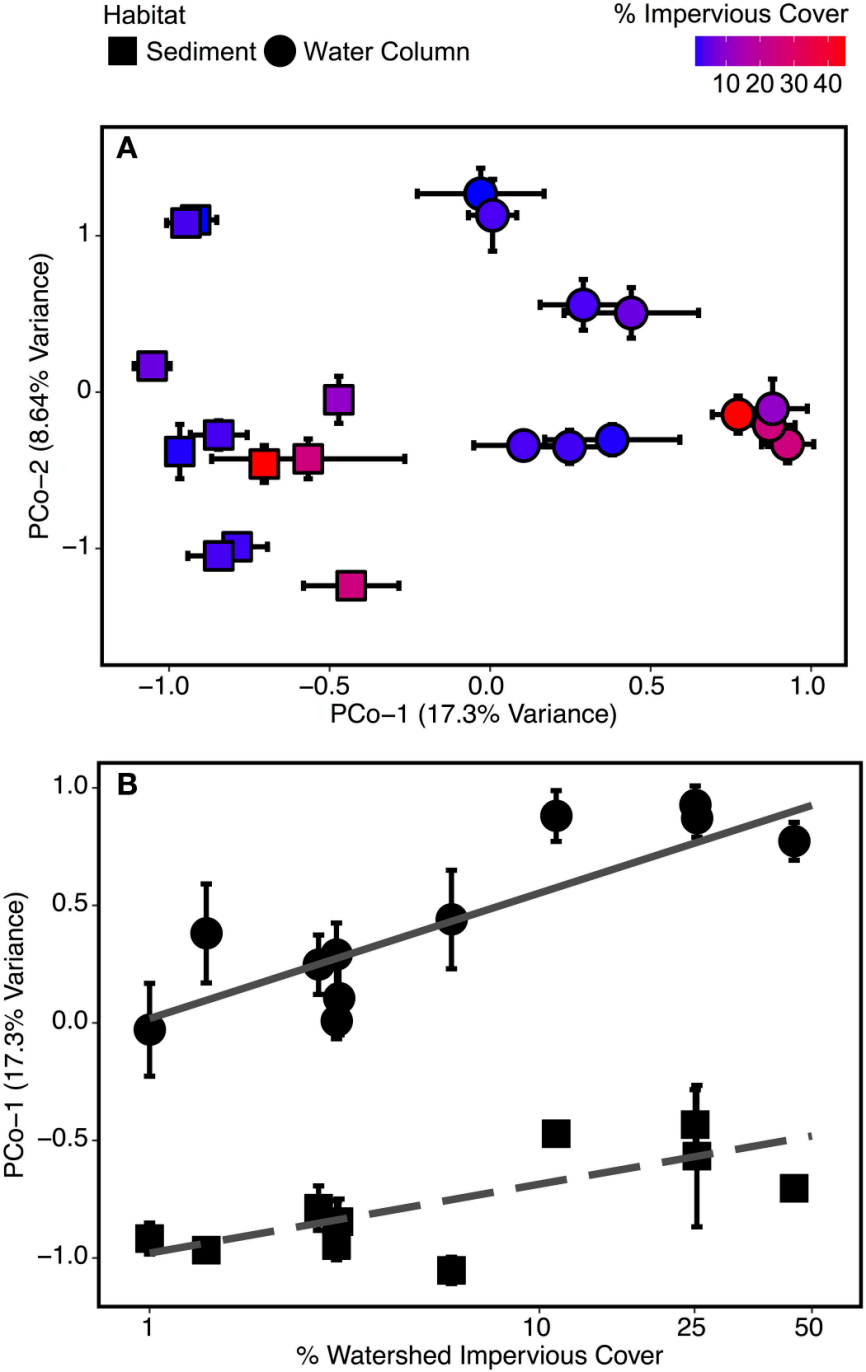
Biplot of **(A)** the first two PCoA scores of microbial OTU Bray-Curtis distances plotted by site and habitat type (sediment and water column) and **(B)** percent watershed impervious cover at a site vs. principal coordinate axis 1 (PCo-1) scores. Each point is identified according to site land cover type (forested and urbanized) and sample habitat (sediment and water column). Error bars represent standard error from repeated measurements taken February 2012-February 2013. OTUs with highest total loadings for principal coordinate (PCo) dimensions 1 and 2 are identified in Table 2.

Bacterial community composition was significantly related to log-transformed watershed percent impervious cover (*r*^2^ = 0.11; *p* < 0.01) and habitat type (*r*^2^ = 0.28; *p* < 0.01) based on PCoA (Figure 4A) and Adonis PERMANOVA of Bray-Curtis distances, with similar patterns observed for weighted and unweighted Unifrac distances (Supplementary Figure 2). The effect of impervious cover was nested within habitat type (Figure 4A) indicating that habitat played the primary role structuring communities. This was confirmed by a significant ANOSIM result (*p* < 0.01), for which the highest R statistics as obtained from habitat (0.78) followed by a combination of habitat and impervious cover (0.74).

Community composition across sample types was separated along the first principal coordinate (PCo-1) axis—the x-axis of Figure 4A. Values below zero were generally from sediment samples and values above zero were generally from water column samples. Urbanized sites also had larger PCo-1 scores than corresponding forested samples, but with a weaker effect than for habitat. The result is the pattern observed in Figure 4A—sediment and water column samples grouped separately, but the highest scores in each group were from urbanized sites. The three OTUs with the highest PCo-1 scores—and thus most abundant in water column and urbanized samples—were OTU 4 (genus *Polynucleobacter*), OTU 1 (genus *Albidiferax*), and OTU 2 (uncultured Methylococcales clone CABC2E06). Two OTUs, OTU 5 (genus *Crenothrix*), and OTU 10 (Order Rhizobiales), had substantially negative PCo-1 scores and these were representative of sediment and forested samples (Table 2).

**Table 2:** OTUs with highest total loadings for principal coordinate (PCo) dimensions 1 and 2 presented in Figure 4A.

**OTU ID**	**PCo-1 Score**	**PCo-2 Score**	**OTU Taxonomy**
OTU 1	1.213	0.554	Bacteria|Proteobacteria|Betaproteobacteria|Burkholderiales|Comamonadaceae|Albidiferax
OTU 2	0.801	−0.625	Bacteria|Proteobacteria|Gammaproteobacteria|Methylococcales|CABC2E06|NC
OTU 4	1.384	−0.194	Bacteria|Proteobacteria|Betaproteobacteria|Burkholderiales|Burkholderiaceae|Polynucleobacter
OTU 5	−0.496	−0.674	Bacteria|Proteobacteria|Gammaproteobacteria|Methylococcales|Crenotrichaceae|Crenothrix
OTU 10	−0.376	0.393	Bacteria|Proteobacteria|Alphaproteobacteria|Rhizobiales|NC
OTU 12	0.496	0.438	Bacteria|Proteobacteria|Epsilonproteobacteria|Campylobacterales|Helicobacteraceae|Sulfuricurvum
OTU 13	0.148	0.759	Bacteria|Proteobacteria|Gammaproteobacteria|Legionellales|Coxiellaceae|Rickettsiella
OTU 14	0.657	−0.170	Bacteria|Proteobacteria|Betaproteobacteria|Nitrosomonadales|Gallionellaceae|Gallionella
OTU 18	0.409	−0.107	Bacteria|Actinobacteria|Actinobacteria|Frankiales|Sporichthyaceae|Candidatus Planktophila
OTU 19	0.427	−0.063	Bacteria|Bacteroidetes|Cytophagia|Cytophagales|Cytophagaceae|Arcicella

**Figure 5:**
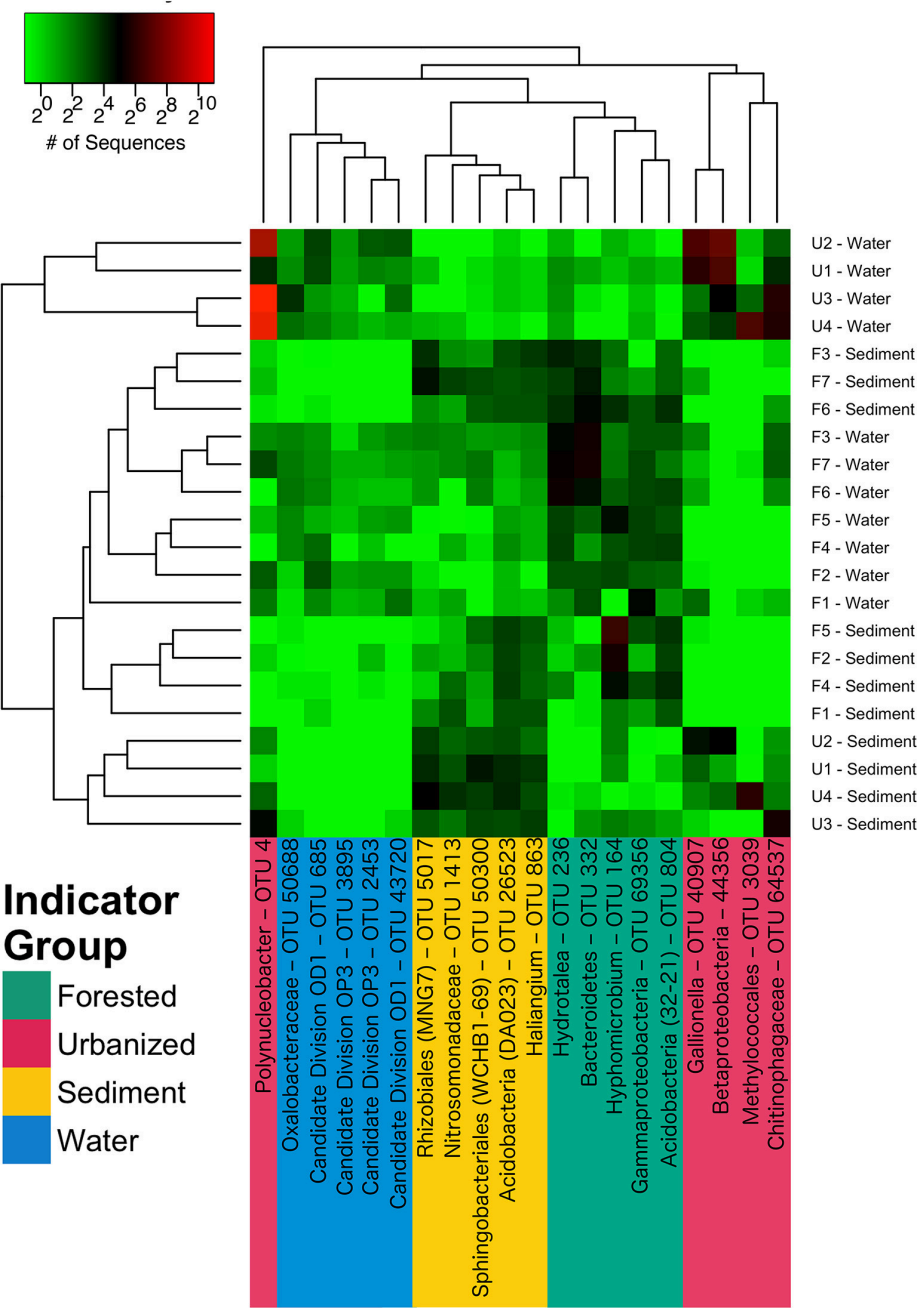
A heatmap of most abundant indicator species for samples from water column, sediment, urbanized sites, and forested sites. Samples and OTUs are arranged according to Bray-Curtis distance cluster analysis. Cell color indicates mean sequence abundance over the course of the study of OTUs by site and habitat type; note that color is on a base 2 logarhithmic scale. OTUs are color-coded according to indicator group: magenta = urbanized, green = forested, blue = water column, and yellow = sediment). Full indicator scores and full taxonomic identification of each OTU is reported in Table 3.

A total of 312 taxa were identified as indicators of water column, sediment, urbanized streams, or forested streams; most (176), were associated with urbanized sites including the five indicator OTUs with the greatest sequence abundance (Figure 5). Indicators of urbanization had very high sequence abundance in urbanized samples, but were rare in forested samples (Figure 5). The most abundant indicator for urbanized sites belonged to the genus *Polynucleobacter* (OTU 4), while the most abundant indicator for forested sites was from the genus *Hyphomicrobium* (OTU 164).

**Table 3:** The most abundant taxa from each of four indicator groups as identified by indicator species analysis.

**Indicator group**	**OTU ID**	**Indicator value**	***p*-value**	**Taxonomy**	**Mean # Seq**.	**Mean # Seq**.	**Mean # Seq**.	**Mean # Seq**.
					**Forested Sed**.	**Forested WC**	**Urb. Sed**.	**Urb. WC**
Sediment	OTU_26523	0.887	0.015	Bacteria/Acidobacteria/Acidobacteria/DA023/NC/NC	9.5	1.8	13.6	1.1
Sediment	OTU_863	0.877	0.015	Bacteria/Proteobacteria/Deltaproteobacteria/Myxococcales/Haliangiaceae/Haliangium	8.0	1.9	9.9	0.6
Sediment	OTU_1413	0.839	0.01	Bacteria/Proteobacteria/Betaproteobacteria/Nitrosomonadales/Nitrosomonadaceae/NC	4.9	2.2	8.9	0.4
Sediment	OTU_50300	0.824	0.02	Bacteria/Bacteroidetes Sphingobacteriia/Sphingobacteriales/WCHB1-69/NC	4.8	1.8	13.5	0.6
Sediment	OTU_5017	0.813	0.01	Bacteria/Proteobacteria Alphaproteobacteria Rhizobiales/MNG7/NC	6.8	2.1	18.9	0.8
Forested	OTU_804	0.850	0.005	Bacteria/Acidobacteria/Acidobacteria/32-21/NC/NC	10.9	5.2	0.9	0.5
Forested	OTU_69356	0.835	0.005	Bacteria/Proteobacteria/Gammaproteobacteria/NC/NC/NC	4.8	12.6	1.0	1.5
Forested	OTU_164	0.800	0.01	Bacteria/Proteobacteria/Alphaproteobacteria/Rhizobiales/Hyphomicrobiaceae/Hyphomicrobium	25.6	4.9	2.5	0.8
Forested	OTU_332	0.789	0.015	Bacteria/Bacteroidetes/NC/NC/NC/NC	9.9	26.1	0.8	0.9
Forested	OTU_236	0.756	0.005	Bacteria/Bacteroidetes/Sphingobacteriia/Sphingobacteriales/Chitinophagaceae/Hydrotalea	6.9	18.3	0.3	2.4
Urbanized	OTU_4	0.892	0.005	Bacteria/Proteobacteria/Betaproteobacteria/Burkholderiales/Burkholderiaceae/Polynucleobacter	0.6	5.9	8.4	948.8
Urbanized	OTU_64537	0.868	0.005	Bacteria/Bacteroidetes/Sphingobacteriia/Sphingobacteriales/Chitinophagaceae/none	0.5	3.7	11.6	32.9
Urbanized	OTU_40907	0.825	0.015	Bacteria/Proteobacteria/Betaproteobacteria/Nitrosomonadales/Gallionellaceae/Gallionella	0.6	2.6	7.9	48.3
Urbanized	OTU_44356	0.654	0.005	Bacteria/Proteobacteria/Betaproteobacteria/NC/NC/NC	0.1	0.2	8.8	84.4
Urbanized	OTU_3039	0.460	0.005	Bacteria/Proteobacteria/Gammaproteobacteria/Methylococcales/NC/NC	0.1	0.5	19.2	31.5
Water Column	OTU_685	0.856	0.02	Bacteria/Candidate Division OD1 /NC/NC/NC/NC	0.4	4.6	0.1	6.9
Water Column	OTU_43720	0.761	0.01	Bacteria/Candidate Division OD1/ NC/NC/NC/NC	0.1	3.2	0.2	4.4
Water Column	OTU_2453	0.689	0.02	Bacteria/Candidate Division OP3/ NC/NC/NC/NC	0.3	2.2	0.0	3.2
Water Column	OTU_50688	0.631	0.03	Bacteria/Proteobacteria/Betaproteobacteria/Burkholderiales/Oxalobacteraceae/NC	0.4	2.4	0.2	5.8
Water Column	OTU_3895	0.573	0.005	Bacteria/Candidate Division OP3/ NC/NC/NC/NC	0.1	1.6	0.0	2.2

*In the taxonomy column, “NC” indicates that a particular taxonomic level is not classified. Four indicator groups were reported: sediment (indicator of both forested and urbanized sediment samples), water column (indicator of forested and urbanized water column samples), forested (indicator of sediment and water column forested samples), and urbanized (indicator of sediment and water column urbanized samples)*.

**Figure 6:**
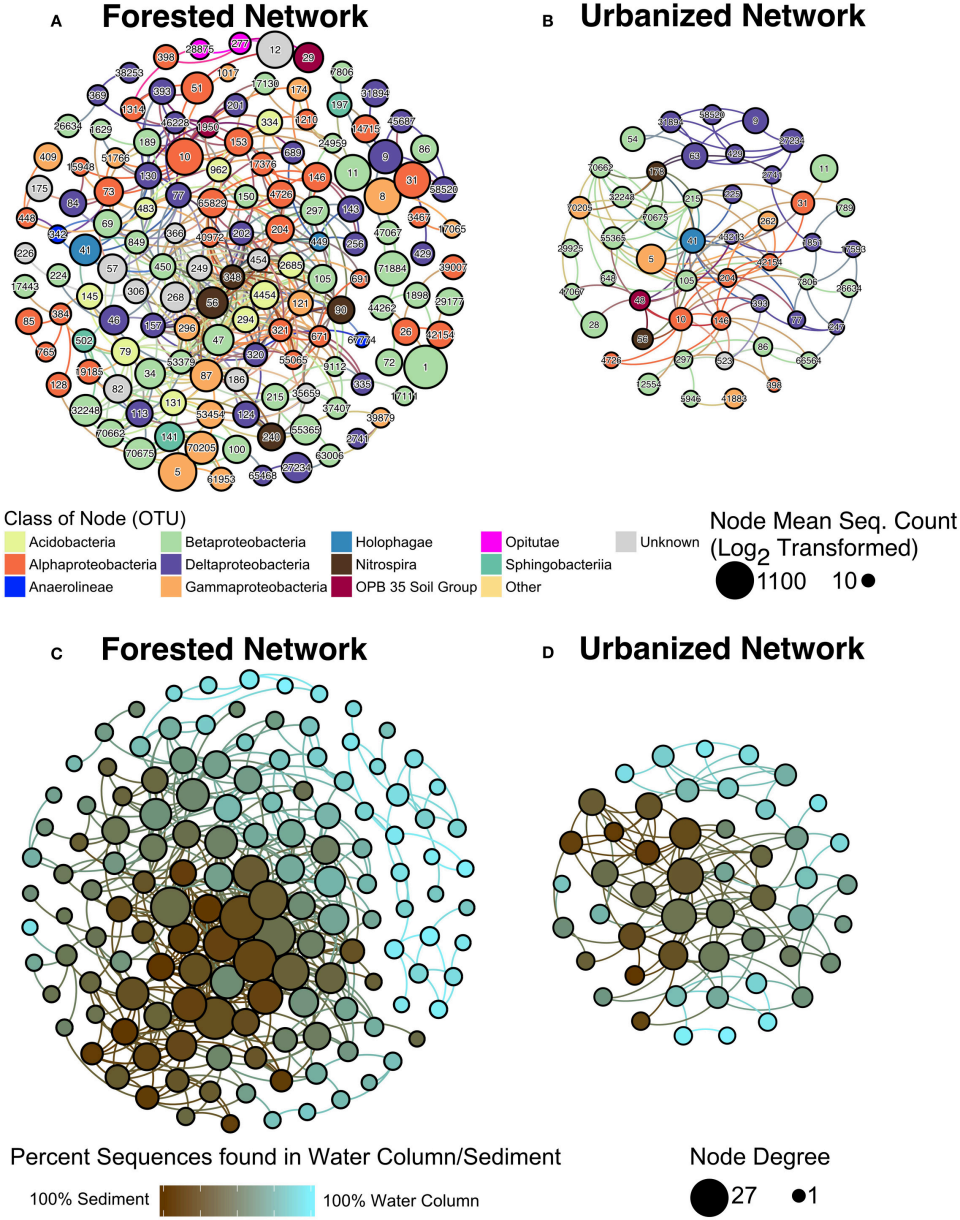
Microbial co-occurrence networks from **(A)** forested, **(B)** urbanized watersheds. Each node is color coded according to its taxonomic class and the size of the node represents the Log_2_ of the mean number of sequences across all samples linked to the OTU identified. The **(C)** forested and **(D)** urbanized networks were replotted with node color indicating the proportion of sequences linked to water column vs. sediment samples for that OTU and node size indicates degree, which is the number of vertices that connect to that node. The taxonomic identity and network statistics for each OTU ID indicated in panels **(A,B)** is included in Supplementary Table 1.

Co-occurrence networks further demonstrated that microbial relationships differed significantly between sites with forested and urbanized catchments (Figure 6; *p* < 0.001). Betaproteobacteria were abundant with high centrality and node degree in both forested and urbanized networks (Figure 6, Supplementary Table 1). By contrast, OTUs from the class Acidobacteria were only dominant in the forested network.

The urbanized network was smaller and less connected than the forested network (Table 4, Supplementary Table 1). Fewer OTUs were present in urbanized networks compared to forested networks and the average node degree—the number of significant co-occurrence events per taxon (Freeman, 1978)—was substantially lower in urbanized (2.64) than forested (3.81) samples. Forested networks also displayed higher transitivity (a measure of connectedness within clusters) than urbanized networks (Table 4).

**Table 4:** Network statistics of the microbial co-occurrence networks (Figure 6).

**Site watershed type**	**# of Nodes**	**# of Edges**	**Avg. node degree**	**Avg. path length**	**Transitivity**	**Modularity**
Forested	142	541	3.81	4.58	0.450	0.457
Urbanized	50	132	2.64	3.01	0.427	0.405

*Transitivity and modularity are normalized. Complete network files are included in Supplementary Datasheet 3*.

**Figure 7:**
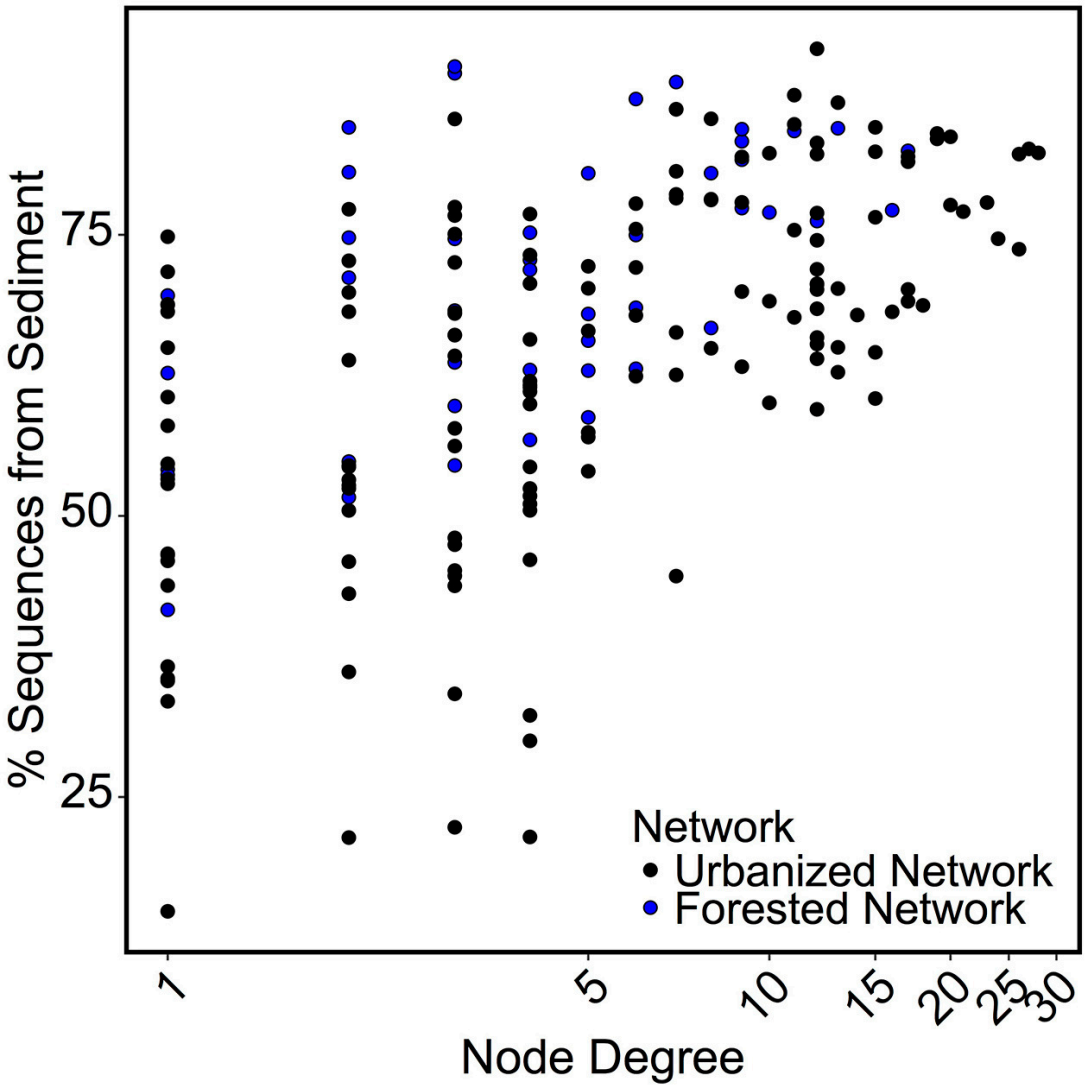
Scatterplot comparing node degree to the percent of sequences found in sediment samples for the OTU represented by that node. A significant positive correlation (*p* < 0.01) was found across both networks.

Networks were analyzed to identify characteristics of OTUs that are most likely to be keystone taxa, specifically those with high centrality and node degree (Berry and Widder, 2014; Widder et al., 2014). Sequence count of an OTU was not correlated to the node degree of that OTU. Instead, we found for both the forested and urbanized co-occurrence networks that node degree of an OTU was positively correlated with the percent of sequences from that OTU that were found in sediment samples (*p* < 0.001; Figure 7).

### Environmental factors and community composition

A significant habitat-type interaction was found when PCo-1 scores were compared to watershed impervious cover using ANCOVA [*F*_(1, 18)_ = 4.57; *p* < 0.05]. Both water column [F_(1, 9)_ = 25.81; *p* < 0.001] and sediment [F_(1, 9)_ = 9.96; *p* < 0.05] samples were significantly related to log-transformed percent impervious cover, though the slope and strength of this relationship differed between the two sample types (Figure 4B). The greater slope for the water column relationship indicated that water column communities were more responsive to changes in impervious cover than sediment communities.

**Figure 8:**
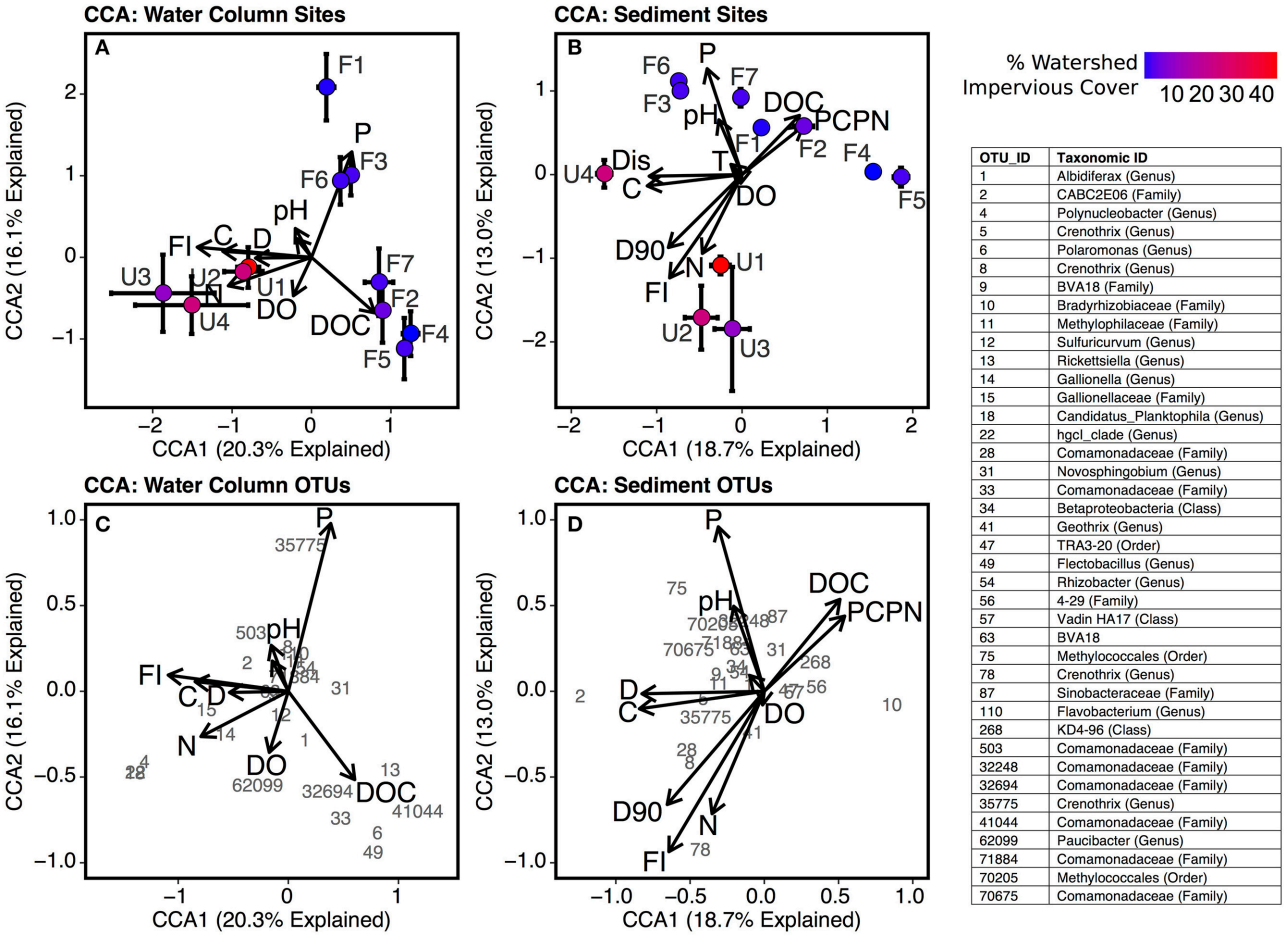
Biplots of canonical correspondence analysis (CCA) scores for environmental factors (indicated by arrows) and scores for each site using **(A)** water column samples and **(B)** sediment samples. Sites are plotted according to mean CCA scores with error bars representing standard error of the mean across replicate samples. Biplots with environmental factors and top OTUs (identified by number) in **(C)** water column samples and **(D)** sediment samples are also included. Factors are abbreviated as follows: Sediment C:N (PCPN), orthophosphate (P), sediment D90 (d90), conductivity **(C)**, discharge **(D)**, dissolved organic carbon concentration (DOC), fluorescence index (FI), temperature (T), and total dissolved nitrogen (TDN). For clarity purposes, the factor loading arrow for C:N of suspended particulates is unlabeled in panels **(A,C)**.

The canonical correlation analysis (CCA) linking bacterial community composition to environmental conditions showed that community composition was more strongly related to environmental variables in sediment (CCA *r*^2^ = 0.53; Figure 8A) than water column samples (*r*^2^ = 0.40; Figure 8B). Bacterial communities in urbanized stream sediments and water column were associated with higher discharge, FI, TDN, and conductivity, while communities in forested stream sediments and water column were positively correlated with ortho-phosphate and DOC concentrations. Several water column OTUs were correlated with high total dissolved nitrogen levels including *Polynucleobacter* (OTU 4), *Gallionella* (OTU 14), and *Gallionellaceae* (OTU 15), genus *Candidatus Planktophila* (OTU 18), and from the hgcl clade of the family *Sporichthyaceae* (OTU 22). Sediment communities in urbanized streams were positively linked with sediment particle size (D90), and sediment communities in forested streams were positively linked to sediment C:N and pH.

## Discussion

Bacterial community composition in both sediment and water column was significantly correlated with urbanization, as measured by watershed impervious cover. However, the underlying factors driving microbial community response to urbanization were different for water column vs. sediment. Water column communities showed a stronger connection to watershed urbanization than sediment communities, providing evidence that water column communities may have been more strongly influenced by dispersal and growth of organisms from sites of anthropogenic disturbance on the landscape. By contrast, sediment communities showed a stronger connection to environmental conditions within the stream reach sampled including discharge, conductivity, and nutrient levels, suggesting that species sorting had a greater impact on sediment bacterial communities. While we do not have direct evidence of the ultimate source of water column microbial taxa, our finding that microbial community composition in the water column is more tightly linked to landscape conditions than sediment communities is consistent with previous studies suggesting that most bacteria in headwater streams may originate from upstream watersheds, however the taxonomic composition of these communities is subsequently modified by environmental sorting within the streams (Crump et al., 2012; Adams et al., 2014; Souffreau et al., 2014). In contrast to these patterns in beta-diversity, microbial alpha-diversity was not strongly connected to urbanization, but the microbial co-occurrence network for urbanized sites was smaller and less connected than for forested samples. We hypothesize that urbanized sites are subjected to an increased magnitude or frequency of disturbance events that is driving a loss of keystone-like taxa in urbanized streams.

Microbial beta-diversity was significantly related to urbanization, measured as log-transformed percent watershed impervious cover, regardless of the habitat type—water or sediment. Urbanized stream sites included taxa that have been associated with eutrophic conditions, human activity, and other impacts of urban infrastructure. *Polynucleobacter* was identified as an indicator of urbanized streams and was highly abundant in those samples—accounting for 1.65% of sequences in urbanized stream samples compared to only 0.58% of sequences in forested stream samples. This OTU is a genus of picobacteria that has been linked to high levels of planktonic autotrophic activity and warm temperatures and is more often associated with larger rivers rather than headwater streams (Hahn, 2003; Boenigk et al., 2004; Wu and Hahn, 2006). Hence, the presence of *Polynucleobacter* suggests the establishment of a more planktonic microbial community, possibly in response to increased nitrogen levels in these streams. Other taxa identified as indicators of urbanized streams included *Albidiferax* species, which were found to be ubiquitous in groundwater contaminated with tetraclorethene (Kotik et al., 2013), and *Gallionella* sp., which are responsible for corrosion in water distribution systems (Ridgway et al., 1981). Thus, the manner in which urbanization shapes stream bacterial communities can be multifold—representing both local environmental impacts and regional processes. Our data suggest that nutrient inputs favor development of a eutrophic community within the stream environment, while infrastructure such as leaky pipes within a watershed deliver novel taxa directly to urbanized streams.

The effects of urbanization were also demonstrated by the CCA, which showed that bacterial communities at forested sites were associated with higher phosphorus levels, while communities at urbanized sites were linked to higher nitrogen levels. This analysis provided further evidence that elevated nitrogen in urbanized streams fuels a shift toward planktonic communities. According to the CCA analysis, *Polynucleobacter (OTU 4)* and *Candidatus Planktophila limnetica* (OTU 18) were both positively associated with higher levels of total dissolved nitrogen in the water column (Figure 8C). The latter taxon is a planktonic actinobacterium (Jezbera et al., 2009) that, like *Polynucleobacter*, was also highly abundant in urbanized samples. An OTU from the genus *Paucibacter* (OTU 62099) was also linked to higher nitrogen levels. Species from this genus degrade toxic peptides produced by cyanobacteria and may be linked to phytoplankton blooms (Rapala et al., 2005). Forested and urbanized stream sites were located at similar positions within the stream network and had similar water velocity and discharge during baseflow conditions, yet urbanized streams had a large number of taxa that are typically associated with water bodies with relatively high water residence times. Increased nutrient levels with urbanization have been linked to increased stream primary production (Alberts et al., 2017) and CCA revealed a link between “planktonic” taxa and higher nitrogen levels (Figure 8). Thus, we argue that increased nutrient levels in combination with a more open canopy at urbanized sites may be stimulating primary production and the rapid growth of a community that would otherwise not be able to develop in a headwater streams with low hydrologic residence times. We acknowledge that the implications of the link between planktonic and nitrogen levels is limited by the observational nature of this study and recommend that experiments under controlled conditions be conducted to evaluate the correlation identified here.

In contrast to urbanized streams, a number of indicator taxa for forested streams were methanotrophic or methylotrophic. The top indicator for forested streams was an OTU from the genus *Hyphomicrobium*—which is composed entirely of facultative methylotrophs (Scheulderman-Suylen and Kuenen, 1985; Rissanen et al., 2016). *Rhizobiales* (OTU 23477) and *Crenothrix* (OTU 5) were two additional taxa linked to forested streams that are likely methanotrophic (Stoecker et al., 2006; Dieser et al., 2014). By contrast, only one top indicator of urbanized streams—an OTU from the order Methylococcales—was identified as methanotrophic (Bowman, 2005; Kato et al., 2013). Previous work in these systems (Febria et al., 2015) and elsewhere (Beck et al., 2013) has demonstrated a strong link between anaerobic methanogens and methane-oxidizing methylotrophs and methanotrophs. Considering this evidence, the relatively high diversity of methane-consuming taxa in forested samples suggests that forested stream microbial communities are structured by inputs from groundwater and the hyporheic zone. Though we lack data on the source of water in urbanized vs. forested streams, the difference in community composition between the two stream types suggests a switch from deep to shallow water flowpaths, suggesting that perhaps watershed urbanization disconnects headwater streams from groundwater supplies.

Forested streams also supported a variety of denitrifying microbes including *Hyphomicrobium* and *Rhizobiales* spp., and had high numbers of nitrifiers including *Nitrospira* which oxidizes nitrite and supports anaerobic ammonia oxidizing (anammox) bacteria that convert ammonia to dinitrogen gas (Daims et al., 2001; Park et al., 2015). In contrast, these taxa were not favored in urbanized streams, providing further evidence of decreased nitrogen removal capacity in urbanized streams. This finding is consistent with other studies that have demonstrated a loss of stream denitrifiers in urbanized streams (Perryman et al., 2011b; Wang et al., 2011).

Microbial co-occurrence networks provide a way to assess the degree to which microbial communities are integrated, and to identify taxa that are important drivers of these relationships (Barberán et al., 2011). Interactions can include competition for resources, parasitism, or mutualistic interactions such as co-metabolism (Parter et al., 2007; Faust et al., 2012). The forested co-occurrence network was significantly larger than the urbanized network, indicating a loss of keystone-like microbial taxa in urbanized streams (Berry and Widder, 2014; Figure 6, Supplementary Datasheet 3). The urbanized network also demonstrated lower average path length and transitivity than the forested network, indicating less interaction across taxa in urbanized microbial communities (Faust et al., 2012).

Microbial co-occurrence network structure is responsive to environmental disturbance (Ruiz-Moreno et al., 2006; Parter et al., 2007), which may explain the difference in size and structure between the forested and urbanized networks. In urbanized streams, environmental conditions (e.g., temperature, hydrology) were likely more variable and more extreme than in forested streams (Nelson and Palmer, 2007; Coleman et al., 2010; Stanley et al., 2010). Previous studies examining the impact of disturbance on microbial co-occurrence networks observed an increase in modularity with increasing disturbance, so long as disturbances were short-term in nature (Ruiz-Moreno et al., 2006; Parter et al., 2007). In this study, modularity was roughly equivalent in urbanized and forested networks, but the number of keystone-like taxa was drastically reduced in the urbanized network. This would be the case if less-adapted taxa become completely inactive due to the long-term nature of urbanization's effect on watersheds. Our measurement of diversity can include taxa that have high sequence abundance, but are functionally unimportant or largely dormant (Shi et al., 2012; Stewart et al., 2012). Thus, these microbes may persist in the environment, but no longer interact with other microbes—in which case these taxa would no longer appear in microbial co-occurrence networks. We propose that the smaller size of the urbanized network reflects a reduction of bacterial taxa that exhibit keystone-like behavior in urbanized streams as some previously well-adapted taxa become less functionally important under urbanized conditions.

For both forested and urbanized networks, OTU node degree was positively correlated with the proportion of sequences for that OTU found in sediment samples (Figure 7). This means that taxa that interact with many other microbial taxa are more likely to be found in sediment than water column environments, providing additional support for the claim that stream sediment communities are more strongly linked to environmental conditions within a stream reach than are water column communities. In light of the lines of evidence presented in this study, we argue that the impact of urbanization on bacterial community composition lends evidence to the assertion that different metacommunity processes drive bacterial community composition in sediment vs. water column habitats (Leibold et al., 2004; Souffreau et al., 2014). The relationship with watershed percent impervious cover was stronger for the water column microbes than sediment communities (Figure 4B). On the other hand, sediment bacterial communities were more strongly linked to the environmental milieu—including conductivity, sediment grain size, and sediment carbon content—than nearby water column communities (Figure 8). All of the top indicators of water column microbial communities (Figure 5, Table 3) were taxa that have been found to be associated with groundwater or landscape sources in previous studies. Candidate Division OP3 and OD1 are both linked to groundwater environments (Glöckner et al., 2010; Nelson and Stegen, 2015) and members of *Oxalobacteraceae* are often associated with soils and plants as well as aquatic habitats (Baldani et al., 2014). This provides further evidence that actively dispersing water column microbes are akin to a regional pool of taxa, controlled in large part by mass effects of microbes imported from the surrounding watershed. In relatively stable stream bed sediment environments, species sorting by local environmental factors determines which subset of the dispersed bacterial taxa can become established (Beisner et al., 2006; Souffreau et al., 2014). Our findings suggest that urbanization impacts different parts of the stream environment in fundamentally different ways: water column communities are directly influenced by urbanization while sediment microbial communities are indirectly impacted via alterations to the stream environment and via changes in the inoculating pool of organisms.

In laboratory experiments, Souffreau et al. (2014) demonstrated that bacterial community composition was controlled by species sorting, except in cases where bacteria immigration rates were high. The water column bacteria assemblage in our study streams likely represents such a high-turnover community. All stream sites sampled were located within 250 m of the stream origin. Thus, it is not unreasonable that most bacteria sampled in the water column during baseflow originate within the watershed, which would explain why water column community composition was more strongly related to watershed impervious cover than sediment community composition. Ultimately, as water flows downstream, we anticipate that the selective pressures of the aquatic environment will result in a more typical pelagic community (Adams et al., 2014). In the headwaters, the water column microbial community appears to be controlled by land use activity in the watershed. This correlation between watershed land cover and the dispersing community has an impact on the sediment community as well, causing a cascade of effects that shape the functional capacity of stream ecosystems.

## Conclusions

Bacterial community composition was most strongly related to stream habitat (water vs. sediment) but within a habitat type urbanization was significantly correlated with composition, particularly for the water column community. In contrast, the sediment community composition was more strongly linked to local environmental conditions (e.g., conductivity, sediment C:N). We suggest that these results support using a metacommunity framework to describe how watershed urbanization changes stream bacterial community composition as has been done for microbial biogeography in other aquatic systems (Crump et al., 2007). With such a framework, community composition in the water column is driven by regional factors related to watershed land use, while sediment bacterial community composition is more strongly controlled by local physicochemistry. Bacterial co-occurrence network analysis showed that urbanization can have substantial implications for microbial community interactions—and potentially functional diversity—even though broader measures of diversity showed no differences. The result was an overall loss in the number of keystone-like taxa in urbanized streams, which implies a loss of functional capacity in headwater streams with increasing watershed impervious cover. It is worth noting however, that due to the nature of land use patterns within the Parkers Creek watershed, the urbanized sites were clustered in the western portion of the watershed which could have resulted in some confounding effects. It is very difficult to find watersheds in which urban sites and forested sites are evenly distributed, but future studies may wish to explicitly consider how spatial arrangement of land use influences stream microbial communities.

## Author contributions

JH designed and carried out the work with input from BCC, CF, and MAP. CF assisted with field and laboratory work. BCC assisted with genetic analysis. All authors contributed to drafts and the final manuscript.

### Conflict of interest statement

The authors declare that the research was conducted in the absence of any commercial or financial relationships that could be construed as a potential conflict of interest.
