# Supplementary material for: Watershed Urbanization Linked to Differences in Stream Bacterial Community Composition
Source: Front Microbiol. 2017 Aug 2;8:1452. doi: 10.3389/fmicb.2017.01452 (PMC5539594; doi:10.3389/fmicb.2017.01452)
Supplement: Supplementary file 4 [file DataSheet4.DOCX]

Supplementary Material

**Watershed Urbanization Linked to Changes in Stream Bacterial Community Composition**

**Jacob D. Hosen*, Catherine M. Febria, Byron C. Crump, Margaret A. Palmer**

*** Correspondence:** Jacob Hosen, Yale School of Forestry & Environmental Studies, 21 Sachem Street, New Haven, CT 06511, USA. Phone: 434-409-0569, fax: 203-436-9135, e-mail: [jake.hosen@yale.edu](mailto:jake.hosen@yale.edu).

#
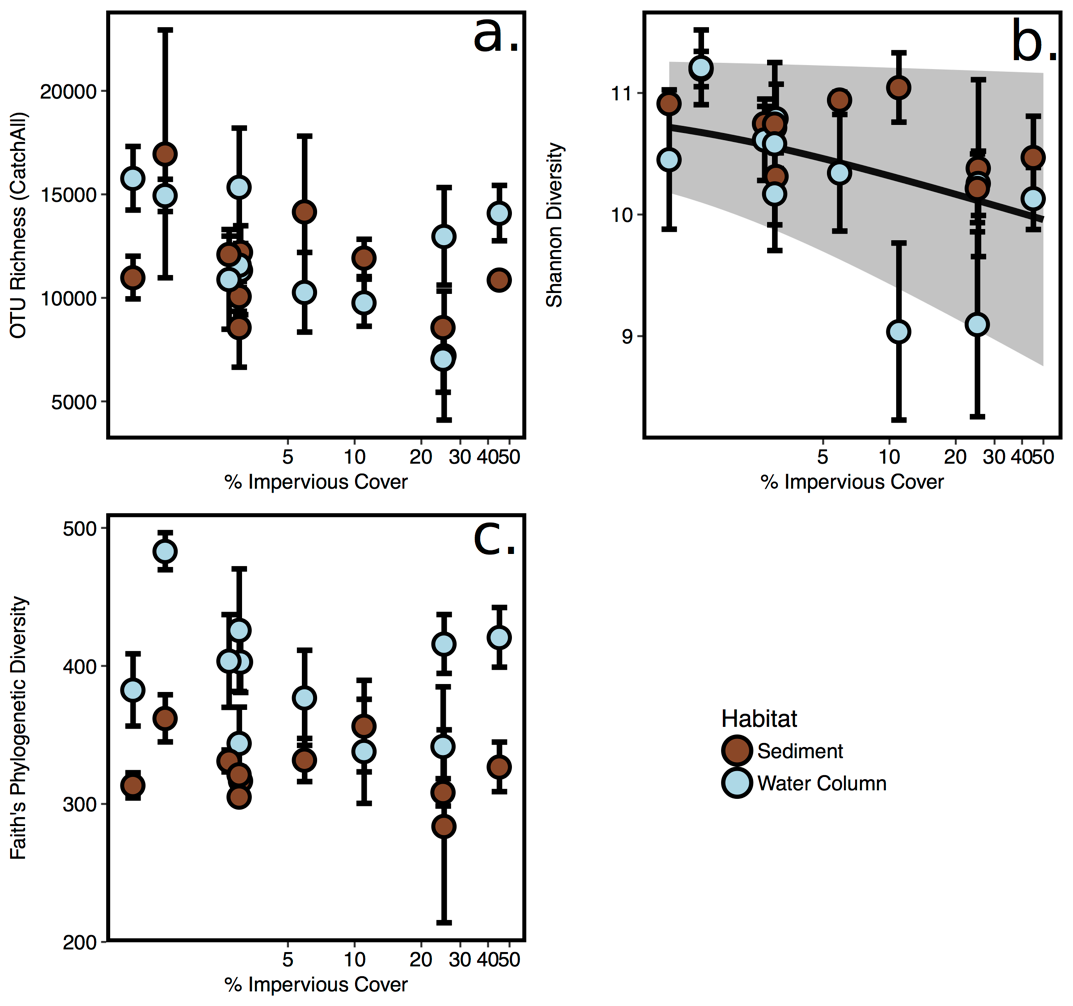
1. Supplementary Figures and Tables

**Supplementary Figure 1:** Plots of stream site watershed % impervious cover compared to three measures of alpha-diversity: (a) OTU richness as measured by CatchAll, (b) Shannon Diversity, and (c) Faith’s Phylogenetic Diversity. Points indicate mean diversity for all samples at a site for a given habitat type. Error bars represent standard error of the mean. A significant negative relationship was found between log-transformed percent impervious cover and Shannon Diversity (b). The regression line and 95% confidence interval for this relationship are included for in the appropriate plot.

*
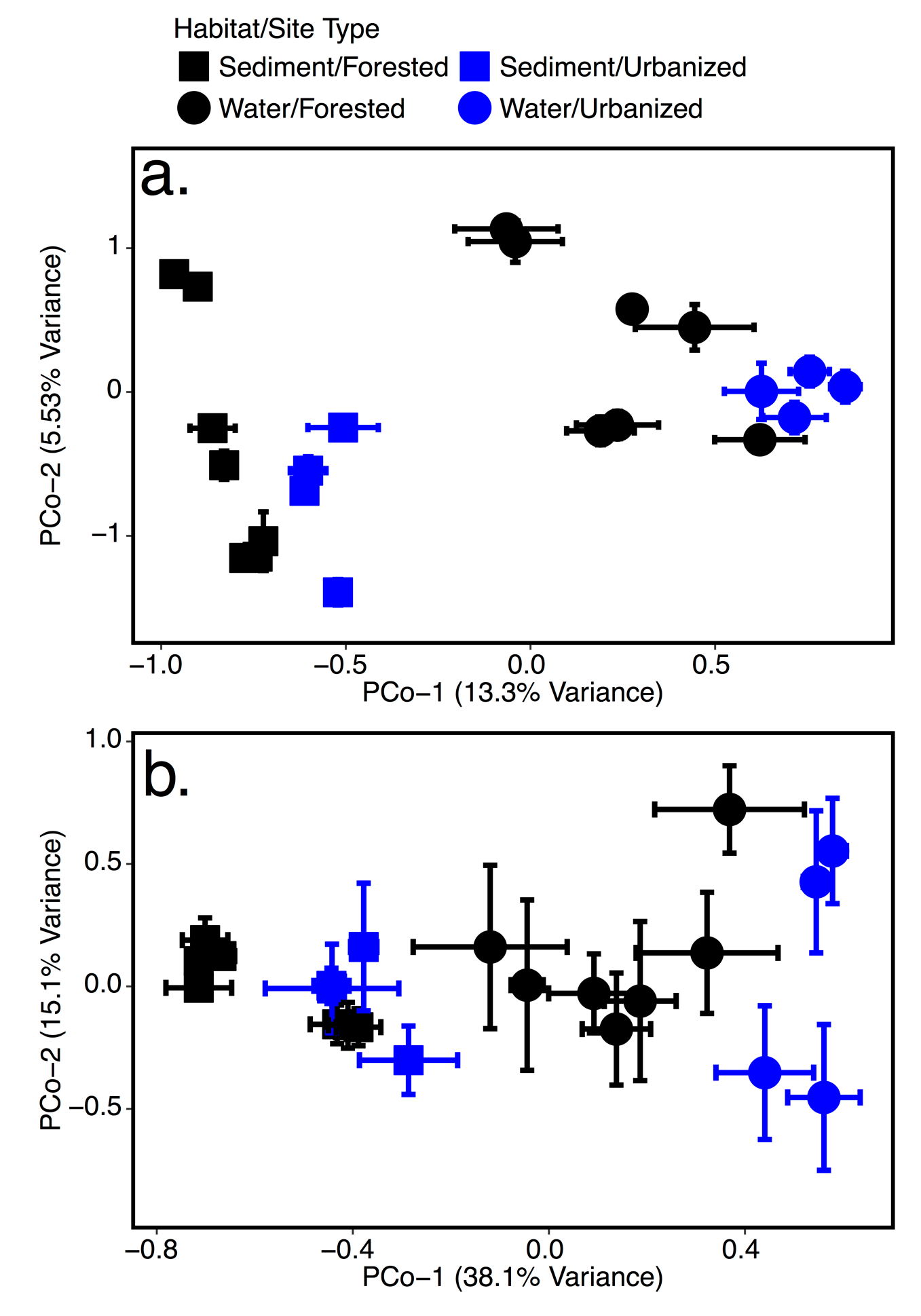
*

**Supplementary Figure 2:** Biplots of (a) the first two PCoA scores of bacterial OTU unweighted Unifrac distances plotted by site and habitat type (sediment and water column) and (b) the first two PCoA scores of bacterial OTU weighted Unifrac distances plotted by site and habitat type (sediment and water column). Each point is identified according to site identity and land cover type (forested and urbanized) and sample habitat (sediment and water column). Error bars represent standard error from repeated measurements.

| **Supplementary Table 1** – A table of the OTUs included in both the Forested and Urbanized microbial co-occurrence networks presented in Figure 6. Each OTU represents an individual node in the network. taxonomic identity, node degree, betweenness, the mean sequences per sample, and percent of sequences obtained from sediment samples are given for each OTU ID. | | | | | | |
| --- | --- | --- | --- | --- | --- | --- |
| **Network** | **OTU ID** | **Degree** | **Betweenness** | **Mean Seq. per Sample** | **% Seq from Sed. Samples** | **Taxonomic Identity** |
| **Forested** | OTU-1 | 3 | 0.0002 | 1119.83 | 22.31 | Bacteria-- Proteobacteria-- Betaproteobacteria-- Burkholderiales-- Comamonadaceae-- Albidiferax |
| **Forested** | OTU-10 | 17 | 0.1105 | 352.00 | 69.08 | Bacteria-- Proteobacteria-- Alphaproteobacteria-- Rhizobiales-- Bradyrhizobiaceae |
| **Forested** | OTU-100 | 4 | 0.0176 | 69.18 | 65.69 | Bacteria-- Proteobacteria-- Betaproteobacteria-- Nitrosomonadales-- Nitrosomonadaceae |
| **Forested** | OTU-1017 | 1 | 0.0000 | 10.99 | 68.76 | Bacteria-- Proteobacteria-- Gammaproteobacteria-- Xanthomonadales-- Sinobacteraceae |
| **Forested** | OTU-105 | 15 | 0.0067 | 77.29 | 64.55 | Bacteria-- Proteobacteria-- Betaproteobacteria |
| **Forested** | OTU-11 | 3 | 0.2841 | 309.29 | 47.44 | Bacteria-- Proteobacteria-- Betaproteobacteria-- Methylophilales-- Methylophilaceae |
| **Forested** | OTU-113 | 12 | 0.0198 | 57.24 | 83.17 | Bacteria-- Proteobacteria-- Deltaproteobacteria-- Myxococcales-- Cystobacteraceae-- Anaeromyxobacter |
| **Forested** | OTU-12 | 1 | 0.0000 | 351.73 | 14.83 | Bacteria-- Proteobacteria-- Epsilonproteobacteria-- Campylobacterales-- Helicobacteraceae-- Sulfuricurvum |
| **Forested** | OTU-121 | 12 | 0.0024 | 41.74 | 70.14 | Bacteria-- Proteobacteria-- Gammaproteobacteria-- Xanthomonadales-- Sinobacteraceae |
| **Forested** | OTU-1210 | 4 | 0.0012 | 18.39 | 51.06 | Bacteria-- Proteobacteria-- Alphaproteobacteria-- Rhizobiales-- Family Incertae Sedis-- Rhizomicrobium |
| **Forested** | OTU-124 | 7 | 0.0156 | 52.89 | 78.26 | Bacteria-- Proteobacteria-- Deltaproteobacteria-- Syntrophobacterales-- Syntrophaceae-- Syntrophus |
| **Forested** | OTU-128 | 2 | 0.0018 | 37.84 | 69.88 | Bacteria-- Proteobacteria-- Alphaproteobacteria-- Rhizobiales-- MNG7 |
| **Forested** | OTU-130 | 16 | 0.0888 | 52.01 | 68.15 | Bacteria-- Proteobacteria-- Deltaproteobacteria-- Desulfuromonadales |
| **Forested** | OTU-131 | 15 | 0.0512 | 46.93 | 82.39 | Bacteria-- Acidobacteria-- Acidobacteria-- DA023 |
| **Forested** | OTU-1314 | 5 | 0.0407 | 34.53 | 57.43 | Bacteria-- Proteobacteria-- Alphaproteobacteria-- Rhizobiales-- Family Incertae Sedis-- Rhizomicrobium |
| **Forested** | OTU-141 | 8 | 0.0193 | 90.83 | 78.17 | Bacteria-- Bacteroidetes-- Sphingobacteriia-- Sphingobacteriales-- Chitinophagaceae-- Terrimonas |
| **Forested** | OTU-143 | 4 | 0.0000 | 47.60 | 61.62 | Bacteria-- Proteobacteria-- Deltaproteobacteria-- Syntrophobacterales-- Syntrophaceae-- Desulfobacca |
| **Forested** | OTU-145 | 5 | 0.0143 | 46.40 | 72.20 | Bacteria-- Acidobacteria-- Acidobacteria-- Order Incertae Sedis-- Order Incertae Sediscross3-- Family Incertae Sedis-- Family Incertae Sediscross4-- Bryobacter |
| **Forested** | OTU-146 | 10 | 0.0105 | 86.12 | 60.06 | Bacteria-- Proteobacteria-- Alphaproteobacteria-- Rhizobiales-- Xanthobacteraceae-- Pseudolabrys |
| **Forested** | OTU-14715 | 1 | 0.0000 | 66.96 | 46.63 | Bacteria-- Proteobacteria-- Alphaproteobacteria-- Sphingomonadales-- Sphingomonadaceae-- Novosphingobium |
| **Forested** | OTU-150 | 9 | 0.0179 | 29.77 | 63.27 | Bacteria-- Proteobacteria-- Betaproteobacteria |
| **Forested** | OTU-153 | 12 | 0.3118 | 70.60 | 65.86 | Bacteria-- Proteobacteria-- Alphaproteobacteria-- Rhizobiales-- Xanthobacteraceae |
| **Forested** | OTU-157 | 12 | 0.0036 | 36.66 | 91.54 | Bacteria-- Proteobacteria-- Deltaproteobacteria-- Syntrophobacterales-- Syntrophobacteraceae |
| **Forested** | OTU-15948 | 3 | 0.0016 | 12.50 | 68.00 | Bacteria-- Proteobacteria-- Alphaproteobacteria-- Rhizobiales-- Hyphomicrobiaceae-- Rhodoplanes |
| **Forested** | OTU-1629 | 3 | 0.0000 | 18.47 | 76.71 | Bacteria-- Proteobacteria-- Betaproteobacteria-- SC-I-84 |
| **Forested** | OTU-17065 | 2 | 0.0142 | 12.47 | 21.39 | Bacteria-- Proteobacteria-- Gammaproteobacteria-- Pseudomonadales-- Pseudomonadaceae-- Pseudomonas |
| **Forested** | OTU-17111 | 1 | 0.0000 | 14.70 | 74.83 | Bacteria-- Proteobacteria-- Betaproteobacteria-- Nitrosomonadales-- Nitrosomonadaceae |
| **Forested** | OTU-17130 | 3 | 0.0002 | 26.14 | 66.09 | Bacteria-- Proteobacteria-- Betaproteobacteria |
| **Forested** | OTU-17376 | 5 | 0.0037 | 31.87 | 57.01 | Bacteria-- Proteobacteria-- Alphaproteobacteria-- Rhizobiales-- Xanthobacteraceae |
| **Forested** | OTU-174 | 4 | 0.0037 | 30.97 | 54.36 | Bacteria-- Proteobacteria-- Gammaproteobacteria-- Pseudomonadales-- Moraxellaceae |
| **Forested** | OTU-17443 | 1 | 0.0000 | 32.18 | 36.60 | Bacteria-- Proteobacteria-- Betaproteobacteria-- Burkholderiales-- Comamonadaceae |
| **Forested** | OTU-175 | 2 | 0.0003 | 49.31 | 68.16 | Bacteria-- Actinobacteria-- Actinobacteria-- Frankiales-- Geodermatophilaceae |
| **Forested** | OTU-186 | 12 | 0.0101 | 31.23 | 82.18 | Bacteria-- Actinobacteria-- Thermoleophilia-- Gaiellales-- Gaiellaceae-- Gaiella |
| **Forested** | OTU-189 | 11 | 0.0119 | 59.52 | 75.42 | Bacteria-- Proteobacteria-- Betaproteobacteria-- Nitrosomonadales-- Nitrosomonadaceae |
| **Forested** | OTU-1898 | 4 | 0.0211 | 47.14 | 21.45 | Bacteria-- Proteobacteria-- Betaproteobacteria-- Burkholderiales-- Comamonadaceae |
| **Forested** | OTU-19185 | 3 | 0.0000 | 37.14 | 72.54 | Bacteria-- Proteobacteria-- Alphaproteobacteria-- Rhizobiales-- MNG7 |
| **Forested** | OTU-1950 | 12 | 0.0205 | 22.47 | 65.28 | Bacteria-- Verrucomicrobia-- OPB35 soil group |
| **Forested** | OTU-197 | 2 | 0.0142 | 37.68 | 36.13 | Bacteria-- Bacteroidetes-- Sphingobacteriia-- Sphingobacteriales-- Chitinophagaceae-- Ferruginibacter |
| **Forested** | OTU-201 | 2 | 0.0000 | 28.92 | 53.21 | Bacteria-- Proteobacteria-- Deltaproteobacteria-- Myxococcales-- Sorangiineae |
| **Forested** | OTU-202 | 20 | 0.1268 | 33.70 | 77.65 | Bacteria-- Proteobacteria-- Deltaproteobacteria-- Syntrophobacterales-- Syntrophobacteraceae |
| **Forested** | OTU-204 | 13 | 0.0096 | 74.60 | 62.78 | Bacteria-- Proteobacteria-- Alphaproteobacteria-- Rhizobiales-- Xanthobacteraceae |
| **Forested** | OTU-215 | 9 | 0.0146 | 55.36 | 69.95 | Bacteria-- Proteobacteria-- Betaproteobacteria-- Nitrosomonadales-- Nitrosomonadaceae |
| **Forested** | OTU-224 | 1 | 0.0000 | 28.04 | 71.71 | Bacteria-- Proteobacteria-- Betaproteobacteria-- Nitrosomonadales-- Nitrosomonadaceae |
| **Forested** | OTU-226 | 1 | 0.0000 | 20.21 | 68.17 | Bacteria-- Bacteroidetes-- Cytophagia-- Cytophagales-- Cytophagaceae-- Flexibacter |
| **Forested** | OTU-240 | 7 | 0.0010 | 65.77 | 86.16 | Bacteria-- Nitrospirae-- Nitrospira-- Nitrospirales-- Nitrospiraceae-- Nitrospira |
| **Forested** | OTU-249 | 13 | 0.0098 | 43.54 | 86.76 | Bacteria-- Spirochaetes-- Spirochaetes-- Spirochaetales-- Spirochaetaceae-- Spirochaeta |
| **Forested** | OTU-24959 | 4 | 0.0056 | 15.34 | 51.77 | Bacteria-- Proteobacteria-- Betaproteobacteria-- Burkholderiales-- Oxalobacteraceae |
| **Forested** | OTU-256 | 4 | 0.0000 | 31.79 | 59.94 | Bacteria-- Proteobacteria-- Deltaproteobacteria-- Syntrophobacterales-- Syntrophaceae-- Desulfomonile |
| **Forested** | OTU-26 | 4 | 0.0347 | 59.18 | 46.09 | Bacteria-- Proteobacteria-- Alphaproteobacteria-- Rhodobacterales-- Rhodobacteraceae-- Rhodobacter |
| **Forested** | OTU-26634 | 1 | 0.0000 | 29.30 | 58.02 | Bacteria-- Proteobacteria-- Betaproteobacteria-- Rhodocyclales-- Rhodocyclaceae-- Propionivibrio |
| **Forested** | OTU-268 | 15 | 0.0048 | 105.98 | 84.56 | Bacteria-- Chloroflexi-- KD4-96 |
| **Forested** | OTU-2685 | 12 | 0.0149 | 46.77 | 68.42 | Bacteria-- Acidobacteria-- Acidobacteria-- DA023 |
| **Forested** | OTU-27234 | 2 | 0.0007 | 102.97 | 52.44 | Bacteria-- Proteobacteria-- Deltaproteobacteria-- Desulfuromonadales-- M20-Pitesti |
| **Forested** | OTU-2741 | 1 | 0.0000 | 18.34 | 60.57 | Bacteria-- Proteobacteria-- Deltaproteobacteria-- Desulfuromonadales |
| **Forested** | OTU-277 | 4 | 0.0284 | 23.77 | 32.26 | Bacteria-- Verrucomicrobia-- Opitutae-- Opitutales-- Opitutaceae-- Opitutus |
| **Forested** | OTU-28875 | 2 | 0.0268 | 18.99 | 45.93 | Bacteria-- Verrucomicrobia-- Opitutae-- Opitutales-- Opitutaceae-- Opitutus |
| **Forested** | OTU-29 | 2 | 0.0148 | 98.39 | 43.08 | Bacteria-- Verrucomicrobia-- OPB35 soil group |
| **Forested** | OTU-29177 | 1 | 0.0000 | 64.08 | 35.55 | Bacteria-- Proteobacteria-- Betaproteobacteria-- Burkholderiales-- Comamonadaceae |
| **Forested** | OTU-294 | 27 | 0.0292 | 48.00 | 82.64 | Bacteria-- Acidobacteria-- Acidobacteria-- DA023 |
| **Forested** | OTU-296 | 17 | 0.0083 | 42.10 | 81.95 | Bacteria-- Proteobacteria-- Gammaproteobacteria-- Order Incertae Sedis-- Order Incertae Sediscross3-- Family Incertae Sedis-- Family Incertae Sediscross4-- Arenicella |
| **Forested** | OTU-297 | 13 | 0.0235 | 81.26 | 70.22 | Bacteria-- Proteobacteria-- Betaproteobacteria-- Nitrosomonadales-- Gallionellaceae |
| **Forested** | OTU-306 | 7 | 0.0219 | 37.89 | 80.65 | Bacteria-- Verrucomicrobia-- S-BQ2-57 soil group |
| **Forested** | OTU-31 | 4 | 0.0142 | 321.17 | 50.49 | Bacteria-- Proteobacteria-- Alphaproteobacteria-- Sphingomonadales-- Sphingomonadaceae-- Novosphingobium |
| **Forested** | OTU-31894 | 1 | 0.0000 | 59.59 | 54.63 | Bacteria-- Proteobacteria-- Deltaproteobacteria-- Desulfuromonadales |
| **Forested** | OTU-320 | 19 | 0.0157 | 32.79 | 83.53 | Bacteria-- Proteobacteria-- Deltaproteobacteria-- Order Incertae Sedis-- Syntrophorhabdaceae-- Syntrophorhabdus |
| **Forested** | OTU-321 | 21 | 0.0208 | 28.04 | 77.06 | Bacteria-- Proteobacteria-- Alphaproteobacteria-- Rhizobiales-- Hyphomicrobiaceae |
| **Forested** | OTU-32248 | 7 | 0.0001 | 132.76 | 86.17 | Bacteria-- Proteobacteria-- Betaproteobacteria-- Burkholderiales-- Comamonadaceae |
| **Forested** | OTU-334 | 5 | 0.0008 | 44.47 | 53.97 | Bacteria-- Acidobacteria-- Acidobacteria-- Candidatus Solibacter |
| **Forested** | OTU-335 | 4 | 0.0000 | 24.47 | 61.99 | Bacteria-- Proteobacteria-- Deltaproteobacteria-- Desulfobacterales-- Desulfobulbaceae |
| **Forested** | OTU-34 | 12 | 0.0209 | 107.38 | 76.94 | Bacteria-- Proteobacteria-- Betaproteobacteria |
| **Forested** | OTU-342 | 4 | 0.0054 | 12.50 | 70.67 | Bacteria-- Chloroflexi-- Anaerolineae-- Anaerolineales-- Anaerolineaceae |
| **Forested** | OTU-3467 | 1 | 0.0000 | 20.56 | 33.51 | Bacteria-- Proteobacteria-- Alphaproteobacteria-- Sphingomonadales-- Sphingomonadaceae-- Novosphingobium |
| **Forested** | OTU-348 | 28 | 0.0601 | 36.12 | 82.28 | Bacteria-- Nitrospirae-- Nitrospira-- Nitrospirales-- 039-6A2 |
| **Forested** | OTU-35659 | 12 | 0.0420 | 16.77 | 70.58 | Bacteria-- Bacteroidetes-- Cytophagia-- Cytophagales-- Cytophagaceae-- Flexibacter |
| **Forested** | OTU-366 | 11 | 0.0077 | 18.01 | 84.82 | Bacteria-- Actinobacteria-- Acidimicrobiia-- Acidimicrobiales-- TM24 |
| **Forested** | OTU-369 | 3 | 0.0283 | 19.26 | 66.07 | Bacteria-- Proteobacteria-- Deltaproteobacteria-- Myxococcales-- Polyangiaceae-- Sorangium |
| **Forested** | OTU-37407 | 5 | 0.0014 | 24.24 | 66.45 | Bacteria-- Proteobacteria-- Betaproteobacteria-- Nitrosomonadales-- Nitrosomonadaceae |
| **Forested** | OTU-38253 | 1 | 0.0000 | 18.49 | 68.81 | Bacteria-- Proteobacteria-- Deltaproteobacteria-- Desulfobacterales-- Desulfobulbaceae-- Desulfobulbus |
| **Forested** | OTU-384 | 6 | 0.0519 | 22.58 | 72.10 | Bacteria-- Proteobacteria-- Alphaproteobacteria-- Caulobacterales-- Hyphomonadaceae-- Woodsholea |
| **Forested** | OTU-39007 | 1 | 0.0000 | 38.89 | 52.86 | Bacteria-- Proteobacteria-- Alphaproteobacteria-- Rhodobacterales-- Rhodobacteraceae |
| **Forested** | OTU-393 | 7 | 0.0088 | 50.10 | 62.54 | Bacteria-- Proteobacteria-- Deltaproteobacteria-- Desulfuromonadales |
| **Forested** | OTU-398 | 1 | 0.0000 | 38.58 | 46.51 | Bacteria-- Proteobacteria-- Alphaproteobacteria-- Rhodospirillales-- Rhodospirillaceae |
| **Forested** | OTU-39879 | 3 | 0.0142 | 16.90 | 56.21 | Bacteria-- Proteobacteria-- Gammaproteobacteria-- Methylococcales |
| **Forested** | OTU-409 | 1 | 0.0000 | 71.07 | 64.96 | Bacteria-- Proteobacteria-- Gammaproteobacteria-- Xanthomonadales-- Sinobacteraceae |
| **Forested** | OTU-40972 | 13 | 0.0066 | 15.90 | 64.99 | Bacteria-- Proteobacteria-- Alphaproteobacteria-- Rhizobiales-- Bradyrhizobiaceae |
| **Forested** | OTU-41 | 5 | 0.0126 | 129.71 | 70.24 | Bacteria-- Acidobacteria-- Holophagae-- Holophagales-- Holophagaceae-- Geothrix |
| **Forested** | OTU-42154 | 3 | 0.0142 | 60.56 | 44.68 | Bacteria-- Proteobacteria-- Alphaproteobacteria-- Sphingomonadales-- Sphingomonadaceae-- Novosphingobium |
| **Forested** | OTU-429 | 1 | 0.0000 | 31.18 | 53.28 | Bacteria-- Proteobacteria-- Deltaproteobacteria-- Desulfuromonadales |
| **Forested** | OTU-44262 | 4 | 0.0956 | 35.19 | 30.00 | Bacteria-- Proteobacteria-- Betaproteobacteria-- Burkholderiales-- Comamonadaceae |
| **Forested** | OTU-4454 | 26 | 0.0256 | 78.00 | 73.72 | Bacteria-- Acidobacteria-- Acidobacteria-- DA023 |
| **Forested** | OTU-448 | 4 | 0.0149 | 18.83 | 61.06 | Bacteria-- Proteobacteria-- Alphaproteobacteria-- Caulobacterales-- Caulobacteraceae-- Phenylobacterium |
| **Forested** | OTU-449 | 15 | 0.0350 | 14.16 | 60.44 | Bacteria-- Acidobacteria-- Holophagae-- SJA-36 |
| **Forested** | OTU-450 | 24 | 0.1410 | 48.52 | 74.65 | Bacteria-- Proteobacteria-- Betaproteobacteria-- SC-I-84 |
| **Forested** | OTU-454 | 23 | 0.0136 | 24.40 | 77.87 | Archaea-- Euryarchaeota-- Thermoplasmata |
| **Forested** | OTU-45687 | 3 | 0.0000 | 28.80 | 45.14 | Bacteria-- Proteobacteria-- Deltaproteobacteria-- Desulfuromonadales-- BVA8 |
| **Forested** | OTU-46 | 6 | 0.0147 | 91.49 | 67.83 | Bacteria-- Proteobacteria-- Deltaproteobacteria-- Desulfuromonadales |
| **Forested** | OTU-46228 | 11 | 0.0691 | 24.63 | 67.66 | Bacteria-- Proteobacteria-- Deltaproteobacteria-- Desulfuromonadales |
| **Forested** | OTU-47 | 18 | 0.0328 | 120.21 | 68.72 | Bacteria-- Proteobacteria-- Betaproteobacteria-- TRA3-20 |
| **Forested** | OTU-47067 | 3 | 0.2091 | 31.72 | 43.78 | Bacteria-- Proteobacteria-- Betaproteobacteria-- Burkholderiales-- Comamonadaceae-- Limnohabitans |
| **Forested** | OTU-4726 | 12 | 0.0371 | 66.43 | 63.97 | Bacteria-- Proteobacteria-- Alphaproteobacteria-- Rhizobiales-- Xanthobacteraceae |
| **Forested** | OTU-483 | 12 | 0.0455 | 22.26 | 70.64 | Bacteria-- Acidobacteria-- Acidobacteria-- DA023 |
| **Forested** | OTU-5 | 3 | 0.0000 | 438.93 | 75.07 | Bacteria-- Proteobacteria-- Gammaproteobacteria-- Methylococcales-- Crenotrichaceae-- Crenothrix |
| **Forested** | OTU-502 | 6 | 0.0066 | 29.94 | 75.51 | Bacteria-- Bacteroidetes-- Sphingobacteriia-- Sphingobacteriales-- AKYH767 |
| **Forested** | OTU-51 | 4 | 0.0283 | 139.74 | 52.44 | Bacteria-- Proteobacteria-- Alphaproteobacteria-- Rhizobiales-- alphaI cluster |
| **Forested** | OTU-51766 | 4 | 0.0000 | 17.42 | 76.85 | Bacteria-- Proteobacteria-- Gammaproteobacteria-- Xanthomonadales-- Sinobacteraceae |
| **Forested** | OTU-53379 | 19 | 0.0375 | 32.73 | 84.01 | Bacteria-- Proteobacteria-- Betaproteobacteria-- SC-I-84 |
| **Forested** | OTU-53454 | 10 | 0.0323 | 66.01 | 69.10 | Bacteria-- Proteobacteria-- Gammaproteobacteria-- Methylococcales-- Crenotrichaceae-- Crenothrix |
| **Forested** | OTU-55065 | 14 | 0.0129 | 13.59 | 67.87 | Bacteria-- Proteobacteria-- Alphaproteobacteria-- Rhizobiales |
| **Forested** | OTU-55365 | 6 | 0.0510 | 101.47 | 62.42 | Bacteria-- Proteobacteria-- Betaproteobacteria-- Burkholderiales-- Comamonadaceae |
| **Forested** | OTU-56 | 20 | 0.0269 | 132.66 | 83.72 | Bacteria-- Nitrospirae-- Nitrospira-- Nitrospirales-- 4-29 |
| **Forested** | OTU-57 | 10 | 0.0404 | 86.79 | 82.26 | Bacteria-- Bacteroidetes-- vadinHA7 |
| **Forested** | OTU-58520 | 3 | 0.0000 | 41.51 | 48.05 | Bacteria-- Proteobacteria-- Deltaproteobacteria-- Desulfuromonadales |
| **Forested** | OTU-61953 | 3 | 0.0000 | 31.98 | 85.30 | Bacteria-- Proteobacteria-- Gammaproteobacteria-- Methylococcales-- Crenotrichaceae-- Crenothrix |
| **Forested** | OTU-63006 | 3 | 0.0104 | 27.40 | 57.79 | Bacteria-- Proteobacteria-- Betaproteobacteria-- Burkholderiales-- Comamonadaceae |
| **Forested** | OTU-65468 | 2 | 0.0043 | 17.64 | 54.47 | Bacteria-- Proteobacteria-- Deltaproteobacteria-- Desulfuromonadales |
| **Forested** | OTU-65829 | 17 | 0.0227 | 85.07 | 70.14 | Bacteria-- Proteobacteria-- Alphaproteobacteria-- Rhizobiales-- Bradyrhizobiaceae |
| **Forested** | OTU-671 | 15 | 0.0251 | 21.19 | 76.56 | Bacteria-- Proteobacteria-- Alphaproteobacteria-- Rhizobiales-- A0839 |
| **Forested** | OTU-67774 | 2 | 0.0000 | 7.33 | 77.27 | Bacteria-- Chloroflexi-- Anaerolineae-- Anaerolineales-- Anaerolineaceae |
| **Forested** | OTU-689 | 4 | 0.0010 | 26.87 | 73.20 | Bacteria-- Proteobacteria-- Deltaproteobacteria-- GR-WP33-30 |
| **Forested** | OTU-69 | 7 | 0.0020 | 69.87 | 66.32 | Bacteria-- Proteobacteria-- Betaproteobacteria-- SC-I-84 |
| **Forested** | OTU-691 | 3 | 0.0000 | 13.23 | 64.23 | Bacteria-- Proteobacteria-- Alphaproteobacteria-- Rhizobiales-- KF-JG30-B3 |
| **Forested** | OTU-70205 | 6 | 0.0094 | 168.58 | 77.77 | Bacteria-- Proteobacteria-- Gammaproteobacteria-- Methylococcales |
| **Forested** | OTU-70662 | 9 | 0.0117 | 65.41 | 77.88 | Bacteria-- Proteobacteria-- Betaproteobacteria-- Burkholderiales-- Comamonadaceae |
| **Forested** | OTU-70675 | 8 | 0.0066 | 137.33 | 85.32 | Bacteria-- Proteobacteria-- Betaproteobacteria-- Burkholderiales-- Comamonadaceae |
| **Forested** | OTU-71884 | 2 | 0.2136 | 238.46 | 50.49 | Bacteria-- Proteobacteria-- Betaproteobacteria-- Burkholderiales-- Comamonadaceae |
| **Forested** | OTU-72 | 1 | 0.0000 | 55.20 | 35.33 | Bacteria-- Proteobacteria-- Betaproteobacteria-- Rhodocyclales-- Rhodocyclaceae-- Ferribacterium |
| **Forested** | OTU-73 | 12 | 0.0399 | 84.57 | 71.94 | Bacteria-- Proteobacteria-- Alphaproteobacteria-- Rhizobiales-- Methylocystaceae-- Methylosinus |
| **Forested** | OTU-765 | 2 | 0.0000 | 15.74 | 72.69 | Bacteria-- Proteobacteria-- Alphaproteobacteria-- Caulobacterales-- Hyphomonadaceae |
| **Forested** | OTU-77 | 12 | 0.0794 | 42.50 | 74.51 | Bacteria-- Proteobacteria-- Deltaproteobacteria-- Desulfobacterales-- Desulfobacteraceae-- Desulfatirhabdium |
| **Forested** | OTU-7806 | 1 | 0.0000 | 21.98 | 46.01 | Bacteria-- Proteobacteria-- Betaproteobacteria |
| **Forested** | OTU-79 | 17 | 0.1003 | 69.19 | 81.50 | Bacteria-- Acidobacteria-- Acidobacteria-- DA023 |
| **Forested** | OTU-8 | 3 | 0.0559 | 330.48 | 34.18 | Bacteria-- Proteobacteria-- Gammaproteobacteria-- Methylococcales-- Crenotrichaceae-- Crenothrix |
| **Forested** | OTU-82 | 11 | 0.0058 | 72.07 | 87.42 | Bacteria-- Acidobacteria-- RB25 |
| **Forested** | OTU-84 | 3 | 0.0010 | 58.73 | 77.47 | Bacteria-- Proteobacteria-- Deltaproteobacteria-- Myxococcales-- Sorangiineae-- Sandaracinaceae |
| **Forested** | OTU-849 | 7 | 0.0053 | 30.68 | 78.59 | Bacteria-- Proteobacteria-- Betaproteobacteria-- SC-I-84 |
| **Forested** | OTU-85 | 2 | 0.0000 | 50.99 | 63.85 | Bacteria-- Proteobacteria-- Alphaproteobacteria-- Rhizobiales-- A0839 |
| **Forested** | OTU-86 | 1 | 0.0000 | 62.64 | 43.81 | Bacteria-- Proteobacteria-- Betaproteobacteria-- Rhodocyclales-- Rhodocyclaceae-- Georgfuchsia |
| **Forested** | OTU-87 | 26 | 0.1150 | 146.98 | 82.17 | Bacteria-- Proteobacteria-- Gammaproteobacteria-- Xanthomonadales-- Sinobacteraceae |
| **Forested** | OTU-9 | 7 | 0.0966 | 207.93 | 44.65 | Bacteria-- Proteobacteria-- Deltaproteobacteria-- Desulfuromonadales-- BVA8 |
| **Forested** | OTU-90 | 9 | 0.0088 | 64.01 | 81.93 | Bacteria-- Nitrospirae-- Nitrospira-- Nitrospirales-- Nitrospiraceae |
| **Forested** | OTU-9112 | 8 | 0.0027 | 15.58 | 64.91 | Bacteria-- Proteobacteria-- Betaproteobacteria-- Nitrosomonadales-- Nitrosomonadaceae |
| **Forested** | OTU-962 | 12 | 0.0237 | 34.47 | 59.48 | Bacteria-- Acidobacteria-- Acidobacteria-- Candidatus Solibacter |
| **Urbanized** | OTU-10 | 11 | 0.0867 | 126.50 | 84.23 | Bacteria-- Proteobacteria-- Alphaproteobacteria-- Rhizobiales-- Bradyrhizobiaceae |
| **Urbanized** | OTU-105 | 16 | 0.1469 | 70.82 | 77.19 | Bacteria-- Proteobacteria-- Betaproteobacteria |
| **Urbanized** | OTU-11 | 1 | 0.0000 | 325.32 | 54.13 | Bacteria-- Proteobacteria-- Betaproteobacteria-- Methylophilales-- Methylophilaceae |
| **Urbanized** | OTU-12554 | 2 | 0.0000 | 91.99 | 84.55 | Bacteria-- Proteobacteria-- Betaproteobacteria-- Rhodocyclales-- Rhodocyclaceae |
| **Urbanized** | OTU-146 | 12 | 0.2327 | 48.25 | 76.22 | Bacteria-- Proteobacteria-- Alphaproteobacteria-- Rhizobiales-- Xanthobacteraceae-- Pseudolabrys |
| **Urbanized** | OTU-17593 | 3 | 0.0132 | 26.53 | 68.26 | Bacteria-- Proteobacteria-- Deltaproteobacteria-- Desulfuromonadales |
| **Urbanized** | OTU-178 | 9 | 0.2465 | 53.61 | 83.31 | Bacteria-- Nitrospirae-- Nitrospira-- Nitrospirales-- Nitrospiraceae |
| **Urbanized** | OTU-1851 | 5 | 0.0576 | 36.91 | 62.92 | Bacteria-- Proteobacteria-- Deltaproteobacteria-- Desulfuromonadales-- M20-Pitesti |
| **Urbanized** | OTU-204 | 10 | 0.0332 | 41.85 | 76.99 | Bacteria-- Proteobacteria-- Alphaproteobacteria-- Rhizobiales-- Xanthobacteraceae |
| **Urbanized** | OTU-215 | 13 | 0.0588 | 44.06 | 84.47 | Bacteria-- Proteobacteria-- Betaproteobacteria-- Nitrosomonadales-- Nitrosomonadaceae |
| **Urbanized** | OTU-225 | 2 | 0.0000 | 40.43 | 74.75 | Bacteria-- Proteobacteria-- Deltaproteobacteria-- Myxococcales-- Cystobacteraceae-- Anaeromyxobacter |
| **Urbanized** | OTU-247 | 3 | 0.0015 | 21.58 | 74.64 | Bacteria-- Proteobacteria-- Deltaproteobacteria-- Desulfobacterales-- Desulfobulbaceae-- Desulfobulbus |
| **Urbanized** | OTU-262 | 5 | 0.0000 | 50.40 | 80.47 | Bacteria-- Proteobacteria-- Gammaproteobacteria-- Methylococcales-- Methylococcaceae-- Methylomonas |
| **Urbanized** | OTU-26634 | 1 | 0.0000 | 36.88 | 69.60 | Bacteria-- Proteobacteria-- Betaproteobacteria-- Rhodocyclales-- Rhodocyclaceae-- Propionivibrio |
| **Urbanized** | OTU-27234 | 5 | 0.0315 | 133.83 | 65.59 | Bacteria-- Proteobacteria-- Deltaproteobacteria-- Desulfuromonadales-- M20-Pitesti |
| **Urbanized** | OTU-2741 | 3 | 0.0585 | 36.25 | 59.78 | Bacteria-- Proteobacteria-- Deltaproteobacteria-- Desulfuromonadales |
| **Urbanized** | OTU-28 | 2 | 0.0000 | 257.33 | 80.57 | Bacteria-- Proteobacteria-- Betaproteobacteria-- Burkholderiales-- Comamonadaceae |
| **Urbanized** | OTU-297 | 4 | 0.0972 | 39.52 | 62.98 | Bacteria-- Proteobacteria-- Betaproteobacteria-- Nitrosomonadales-- Gallionellaceae |
| **Urbanized** | OTU-29925 | 1 | 0.0000 | 38.81 | 62.70 | Bacteria-- Proteobacteria-- Betaproteobacteria-- Burkholderiales-- Comamonadaceae |
| **Urbanized** | OTU-31 | 6 | 0.0663 | 130.71 | 68.51 | Bacteria-- Proteobacteria-- Alphaproteobacteria-- Sphingomonadales-- Sphingomonadaceae-- Novosphingobium |
| **Urbanized** | OTU-31894 | 5 | 0.0035 | 81.30 | 58.76 | Bacteria-- Proteobacteria-- Deltaproteobacteria-- Desulfuromonadales |
| **Urbanized** | OTU-32248 | 3 | 0.0000 | 76.71 | 89.37 | Bacteria-- Proteobacteria-- Betaproteobacteria-- Burkholderiales-- Comamonadaceae |
| **Urbanized** | OTU-393 | 6 | 0.0254 | 32.60 | 74.98 | Bacteria-- Proteobacteria-- Deltaproteobacteria-- Desulfuromonadales |
| **Urbanized** | OTU-398 | 2 | 0.0000 | 13.91 | 52.71 | Bacteria-- Proteobacteria-- Alphaproteobacteria-- Rhodospirillales-- Rhodospirillaceae |
| **Urbanized** | OTU-41 | 17 | 0.2672 | 186.74 | 82.47 | Bacteria-- Acidobacteria-- Holophagae-- Holophagales-- Holophagaceae-- Geothrix |
| **Urbanized** | OTU-41883 | 1 | 0.0000 | 63.77 | 41.65 | Bacteria-- Proteobacteria-- Gammaproteobacteria-- Methylococcales |
| **Urbanized** | OTU-42154 | 8 | 0.0147 | 28.85 | 80.48 | Bacteria-- Proteobacteria-- Alphaproteobacteria-- Sphingomonadales-- Sphingomonadaceae-- Novosphingobium |
| **Urbanized** | OTU-429 | 4 | 0.0408 | 41.77 | 61.44 | Bacteria-- Proteobacteria-- Deltaproteobacteria-- Desulfuromonadales |
| **Urbanized** | OTU-43213 | 3 | 0.0106 | 16.81 | 68.07 | Bacteria-- Proteobacteria-- Deltaproteobacteria-- Desulfuromonadales |
| **Urbanized** | OTU-47067 | 4 | 0.0456 | 24.74 | 72.77 | Bacteria-- Proteobacteria-- Betaproteobacteria-- Burkholderiales-- Comamonadaceae-- Limnohabitans |
| **Urbanized** | OTU-4726 | 2 | 0.0000 | 24.66 | 71.19 | Bacteria-- Proteobacteria-- Alphaproteobacteria-- Rhizobiales-- Xanthobacteraceae |
| **Urbanized** | OTU-48 | 9 | 0.0969 | 68.46 | 84.39 | Bacteria-- Verrucomicrobia-- OPB35 soil group |
| **Urbanized** | OTU-5 | 8 | 0.0036 | 552.44 | 78.10 | Bacteria-- Proteobacteria-- Gammaproteobacteria-- Methylococcales-- Crenotrichaceae-- Crenothrix |
| **Urbanized** | OTU-523 | 5 | 0.0064 | 34.17 | 67.96 | Bacteria-- Bacteroidetes-- BSV3 |
| **Urbanized** | OTU-54 | 2 | 0.0000 | 169.86 | 54.36 | Bacteria-- Proteobacteria-- Betaproteobacteria-- Burkholderiales-- Comamonadaceae-- Rhizobacter |
| **Urbanized** | OTU-55365 | 9 | 0.0263 | 99.09 | 77.37 | Bacteria-- Proteobacteria-- Betaproteobacteria-- Burkholderiales-- Comamonadaceae |
| **Urbanized** | OTU-56 | 3 | 0.0000 | 74.47 | 89.96 | Bacteria-- Nitrospirae-- Nitrospira-- Nitrospirales-- 4-29 |
| **Urbanized** | OTU-58520 | 1 | 0.0000 | 56.76 | 53.64 | Bacteria-- Proteobacteria-- Deltaproteobacteria-- Desulfuromonadales |
| **Urbanized** | OTU-5946 | 2 | 0.0408 | 26.67 | 51.66 | Bacteria-- Proteobacteria-- Betaproteobacteria-- Nitrosomonadales-- Gallionellaceae-- Candidatus Nitrotoga |
| **Urbanized** | OTU-63 | 6 | 0.1702 | 242.53 | 63.09 | Bacteria-- Proteobacteria-- Deltaproteobacteria-- Desulfuromonadales-- BVA8 |
| **Urbanized** | OTU-648 | 3 | 0.0008 | 18.68 | 63.65 | Bacteria-- Proteobacteria-- Betaproteobacteria-- Nitrosomonadales-- Nitrosomonadaceae |
| **Urbanized** | OTU-66564 | 4 | 0.0153 | 30.14 | 71.89 | Bacteria-- Proteobacteria-- Betaproteobacteria-- Rhodocyclales-- Rhodocyclaceae |
| **Urbanized** | OTU-70205 | 6 | 0.0413 | 99.78 | 87.08 | Bacteria-- Proteobacteria-- Gammaproteobacteria-- Methylococcales |
| **Urbanized** | OTU-70662 | 9 | 0.0216 | 69.24 | 81.68 | Bacteria-- Proteobacteria-- Betaproteobacteria-- Burkholderiales-- Comamonadaceae |
| **Urbanized** | OTU-70675 | 7 | 0.0097 | 139.23 | 88.58 | Bacteria-- Proteobacteria-- Betaproteobacteria-- Burkholderiales-- Comamonadaceae |
| **Urbanized** | OTU-77 | 4 | 0.0097 | 34.58 | 75.19 | Bacteria-- Proteobacteria-- Deltaproteobacteria-- Desulfobacterales-- Desulfobacteraceae-- Desulfatirhabdium |
| **Urbanized** | OTU-7806 | 8 | 0.0888 | 21.82 | 66.71 | Bacteria-- Proteobacteria-- Betaproteobacteria |
| **Urbanized** | OTU-789 | 2 | 0.0041 | 36.47 | 54.83 | Bacteria-- Proteobacteria-- Betaproteobacteria-- Rhodocyclales-- Rhodocyclaceae |
| **Urbanized** | OTU-86 | 4 | 0.0240 | 46.98 | 56.76 | Bacteria-- Proteobacteria-- Betaproteobacteria-- Rhodocyclales-- Rhodocyclaceae-- Georgfuchsia |
| **Urbanized** | OTU-9 | 3 | 0.0000 | 228.18 | 54.49 | Bacteria-- Proteobacteria-- Deltaproteobacteria-- Desulfuromonadales-- BVA8 |

| **Supplementary Table 2 –** Metadata for supplementary datasheet 2. This table contains a list of the variable abbreviations found in supplementary datasheet 2 and corresponding descriptions for each variable. | |
| --- | --- |
| **Abbreviation** | **Description** |
| Site | Site ID |
| Date | Sampling Date |
| Habitat | Sampling habitat group (sediment or water column) |
| Type | Watershed type (forested or urbanized) |
| DOC_mgl | Dissolved organic carbon (DOC) concentration in mg/L |
| TDN_mgl | total dissolved nitrogen concentration (mg L^-1^) |
| hix | Humification index |
| FI | Fluorescence index |
| dissolved_n_to_p | ratio of dissolved inorganic nitrogen to dissolved orthophosphate |
| Nitrate_ugl | Dissolved nitrate concentration (µg L^-1^) |
| Ammonia_ugl | Dissolved ammonia concentration (µg L^-1^) |
| orthophosphate_ugl | Dissolved orthophosphate concentration (µg L^-1^) |
| DON_mgl | Dissolved organic nitrogen (DON) concentration (mg L^-1^) |
| Discharge | Discharge (L s^-1^) |
| Impervious_Hect | Watershed impervious cover (Hectares) |
| Total_Hect | Total watershed surface area (Hectares) |
| temperature | Temperature (°C) |
| spc_cond_uscm | Specific conductivity (µs cm^-1^) |
| do_concentration_mgl | Dissolved oxygen (DO) concentration (mg L^-1^) |
| pH | pH |
| catchall_richness | OTU richness as determined with CatchAll |
| catchall_se | Standard error of OTU richness as measured by CatchAll |
| faiths_phylogenetic | Faith's phyolgenetic diversity |
| shannon_diversity | Shannon diversity |
| pc_pn_s | Ratio of sediment particulate carbon to particulate nitrogen |
| per_particulate_n_s | Sediment % particulate mass as nitrogen |
| per_particulate_c_s | Sediment % particulate mass as carbon |
| pc_mgl_w | Water column particulate carbon (mg L^-1^) |
| pn_mgl_w | Water column particulate nitrogen (mg L^-1^) |
| pc_pn_w | Water column particulate C:N |
| d50 | Sediment d50 |
| d90 | Sediment d90 |
| d75d25 | Ratio sediment D75:D25 |
